# Upper respiratory *Streptococcus pneumoniae* colonization among working-age adults with prevalent exposure to overcrowding

**DOI:** 10.1128/spectrum.00879-24

**Published:** 2024-07-16

**Authors:** Anna M. Parker, Nicole Jackson, Shevya Awasthi, Hanna Kim, Tess Alwan, Anne L. Wyllie, Katherine Kogut, Nina Holland, Ana M. Mora, Brenda Eskenazi, Lee W. Riley, Joseph A. Lewnard

**Affiliations:** 1 Division of Epidemiology, School of Public Health, University of California, Berkeley, California, USA; 2 Division of Infectious Diseases & Vaccinology, School of Public Health, University of California, Berkeley, California, USA; 3 Department of Epidemiology of Microbial Diseases, Yale School of Public Health, New Haven, Connecticut, USA; 4 Center for Environmental Research & Community Health, School of Public Health, University of California, Berkeley, California, USA; 5 Center for Computational Biology, College of Engineering, University of California, Berkeley, California, USA; The Ohio State University College of Dentistry, Columbus, Ohio, USA

**Keywords:** *Streptococcus pneumoniae*, carriage, colonization, epidemiology, qPCR

## Abstract

Most pneumococcal disease occurs among infants and older adults and is thought to be driven by the transmission of *Streptococcus pneumoniae* from young children to these vulnerable age groups. However, pneumococcal disease outbreaks also affect non-elderly adults living or working in congregate, close-contact settings. Little is known about pneumococcal carriage in such populations. From July to November 2020, we collected saliva from low-income adult farmworkers in Monterey County, California, and tested for pneumococcal carriage following culture enrichment via quantitative PCR assays targeting the pneumococcal *lytA* and *piaB* genes. Participants were considered to carry pneumococci if *lytA* and *piaB* cycle threshold values were both below 40. Among 1,283 participants enrolled in our study, 117 (9.1%) carried pneumococci. Carriers tended more often than non-carriers to be exposed to children aged <5 years [odds ratio (OR) = 1.45 (0.95–2.20)] and overcrowding [OR = 1.48 (0.96–2.30) and 2.84 (1.20–6.73), respectively, for participants in households with >2–4 and >4 persons per bedroom vs ≤2 persons per bedroom]. Household overcrowding remained associated with increased risk of carriage among participants not exposed to children aged <5 years [OR = 2.05 (1.18–3.59) for participants living in households with >2 vs ≤2 persons per bedroom]. Exposure to children aged <5 years and overcrowding were each associated with increased pneumococcal density among carriers [*piaB* c*_T_* difference of 2.04 (0.36–3.73) and 2.44 (0.80–4.11), respectively]. While exposure to young children was a predictor of pneumococcal carriage, associations of overcrowding with increased prevalence and density of carriage in households without young children suggest that transmission also occurs among adults in close-contact settings.

**IMPORTANCE:**

Although infants and older adults are the groups most commonly affected by pneumococcal disease, outbreaks are known to occur among healthy, working-age populations exposed to overcrowding, including miners, shipyard workers, military recruits, and prisoners. Carriage of *Streptococcus pneumoniae* is the precursor to pneumococcal disease, and its relation to overcrowding in adult populations is poorly understood. We used molecular methods to characterize pneumococcal carriage in culture-enriched saliva samples from low-income adult farmworkers in Monterey County, CA. While exposure to children in the household was an important risk factor for pneumococcal carriage, living in an overcrowded household without young children was an independent predictor of carriage as well. Moreover, participants exposed to children or overcrowding carried pneumococci at higher density than those without such exposures, suggesting recent transmission. Our findings suggest that, in addition to transmission from young children, pneumococcal transmission may occur independently among adults in overcrowded settings.

## INTRODUCTION

The bacterial pathogen *Streptococcus pneumoniae* (pneumococcus) is a prominent cause of severe invasive infections as well as mucosal conditions such as acute otitis media, sinusitis, and non-bacteremic pneumonia among children and adults (1, 2). Commensal carriage of pneumococci in the upper respiratory tract is the source of transmission and a necessary precursor to pneumococcal diseases (3). Children aged 2–4 years are widely understood to serve as the primary reservoir for pneumococcal transmission. While carriage prevalence has been estimated at 30%–34% in recent studies of United States (US) children (4–6), estimates have spanned 17%–90% across settings with differing socioeconomic status, living conditions, and pneumococcal conjugate vaccine (PCV) access (7–9).

Whereas adults account for most pneumococcal disease burden in the US (10, 11) and may contribute to transmission across age groups, understanding of the prevalence and predictors of adult carriage remains limited (12). Studies employing conventional nasopharyngeal sampling techniques and culture-based identification of pneumococci have yielded low (<5%) estimates of carriage prevalence among adults, although novel molecular approaches enabling pneumococcal carriage detection from saliva have identified ≥20% prevalence of adult carriage in some populations (13–16). Studies employing these higher-sensitivity methods for sampling and detection offer an opportunity to better understand pneumococcal epidemiology among adults, providing insight into risk factors for carriage acquisition and the role of adults in transmission within distinct communities.

Working-age adults exposed to crowded conditions have emerged as an important risk group for pneumococcal disease, with invasive pneumococcal disease outbreaks reported among miners (17), military recruits (18), shipyard workers (19), prisoners (20), displaced migrants (21), residents of homeless shelters (22), and others living or working within close, congregate settings (23). While these observations suggest that pneumococcal transmission may occur among adults under crowded conditions without the primary involvement of young children, prior studies reporting pneumococcal outbreaks in such settings have not investigated underlying carriage dynamics among adults. In the US, agricultural workers may comprise an at-risk group for such transmission due to the prevalent exposure to overcrowding, poor ventilation, and suboptimal hygienic conditions in their housing, transportation, and working environments (24). As part of a prior cross-sectional study among adult farmworkers within California’s Salinas Valley, we collected saliva samples for pneumococcal carriage detection while administering SARS-CoV-2 testing in both clinical and community settings from July to November 2020 (25, 25, 26). We revisited data from this study for secondary analyses aiming to characterize potential risk factors and serotypes associated with adult pneumococcal carriage within the study communities.

## RESULTS

### Enrollment and inclusion

Of 1,309 participants who enrolled in the study, our analyses included 1,283 (1,099 farmworkers within the primary study group and 184 additional individuals who participated in the abridged supplemental study not restricted to farmworkers). We excluded 8 participants who did not meet the eligibility criteria (detailed in Materials and Methods), 3 participants who did not provide a saliva sample, and 15 participants whose saliva specimens did not meet the quality criteria (for reasons including label mismatch, risk of contamination, or insufficient saliva volume). Among the participants included, 566 (44.1% of 1,283) enrolled in clinical settings and 717 (55.9%) enrolled in outreach settings (Table 1; Table S1). Most participants spoke Spanish at home (1,055/1,283; 82.2%), had never attended high school (774/1,282; 60.4%), and lived in households earning <$25,000 annually (622/1,212; 51.3%). As compared to participants recruited in clinical settings, a greater proportion of those recruited via outreach testing were born in the US, had completed high school, lived in apartments or hotels (versus other housing types), and were not concurrently infected with SARS-CoV-2.

**TABLE 1:** Characteristics of the study population, by recruitment venue^*a*^

Characteristics		Participants, no. (%)		
		All	Clinic sample	Outreach sample
		*N* = 1,283	*N* = 566	*N* = 717
Study population				
	Primary study	1,099 (85.7)	551 (97.3)	548 (76.4)
	Supplemental study	184 (14.3)	15 (2.7)	169 (23.6)
Age range (years)				
	18–29	344 (26.8)	145 (25.6)	199 (27.7)
	30–39	306 (23.9)	137 (24.2)	169 (23.6)
	40–49	327 (25.5)	158 (27.9)	169 (23.6)
	≥50	306 (23.9)	126 (22.2)	180 (25.1)
Sex				
	Female	698 (54.4)	304 (53.7)	394 (55.0)
	Male	585 (45.6)	262 (46.3)	323 (45.0)
Country of birth				
	Mexico	1,012 (78.9)	484 (85.5)	528 (73.6)
	United States	226 (17.6)	54 (9.5)	172 (24.0)
	Other	45 (3.5)	28 (4.9)	17 (2.4)
Language spoken at home				
	Spanish	1,055 (82.2)	457 (80.7)	598 (83.4)
	English	118 (9.2)	25 (4.4)	93 (13.0)
	Indigenous language^*b*^	110 (8.6)	84 (14.8)	26 (3.6)
Educational attainment (*N* = 1,282)				
	Never attended school	63 (4.9)	48 (8.5)	15 (2.1)
	Some primary school	460 (35.9)	225 (39.8)	235 (32.8)
	Primary school completed	251 (19.6)	117 (20.7)	134 (18.7)
	Some high school	148 (11.5)	66 (11.7)	82 (11.4)
	High school completed	360 (28.1)	109 (19.3)	251 (35.0)
Annual household income (*N* = 1,212)				
	<25,000	622 (51.3)	286 (53.5)	336 (49.6)
	25,000–34,999	284 (23.4)	113 (21.1)	171 (25.3)
	35,000–49,000	195 (16.1)	88 (16.4)	107 (15.8)
	≥50,000	111 (9.2)	48 (9.0)	63 (9.3)
Washing machine in residence (*N* = 1,099)^*c*^				
	No	408 (37.1)	214 (38.8)	194 (35.4)
	Yes	691 (62.9)	337 (61.2)	354 (64.6)
H-2A visa (*N* = 946)^*c*^				
	No	881 (93.1)	477 (96.0)	404 (90.0)
	Yes	65 (6.9)	20 (4.0)	45 (10.0)
Years living in United States (*N* = 958)^*c*^				
	<5 years	105 (11.0)	59 (11.7)	46 (10.1)
	5–18 years	265 (27.7)	159 (31.6)	106 (23.3)
	≥18 years	588 (61.4)	285 (56.7)	303 (66.6)
Field worker occupation (*N* = 1,089)^*c*^				
	No	270 (24.8)	134 (24.8)	136 (24.8)
	Yes	819 (75.2)	407 (75.2)	412 (75.2)
Workplace setting (*N* = 1,098)^*c*^				
	Indoor and outdoor	260 (23.7)	134 (24.4)	126 (23)
	Outdoor only	838 (76.3)	417 (75.6)	422 (77)
Body mass index (*N* = 1,071)^*c*^				
	<18.5, underweight	4 (0.4)	2 (0.4)	2 (0.4)
	18.5–24.9, normal	186 (17.4)	101 (19.0)	85 (15.7)
	25–29.9, overweight	409 (38.2)	202 (38.0)	207 (38.3)
	30–39.9, obese	427 (39.9)	205 (38.6)	222 (41.1)
	≥40, severely obese	45 (4.2)	21 (4.0)	24 (4.4)
Cigarette smoking (*N* = 1,098)^*c*^				
	Never smoked	891 (81.1)	446 (81)	445 (81)
	Former smoker	158 (14.4)	86 (16)	72 (13)
	Current smoker	49 (4.5)	18 (3.3)	31 (5.7)
Wearing a face covering when in proximity to others outside the home (*N* = 1,099)^*c*^				
	Some of the time, rarely, or never	86 (7.8)	44 (8.0)	42 (7.7)
	All or most of the time	1,013 (92.2)	507 (92.0)	506 (92.3)
Housing type				
	House	597 (46.5)	330 (58.3)	267 (37.2)
	Apartment	576 (44.9)	193 (34.1)	383 (53.4)
	Trailer/mobile home	45 (3.5)	23 (4.1)	22 (3.1)
	Hotel/motel	38 (3.0)	8 (1.4)	30 (4.2)
	Other	27 (2.1)	12 (2.1)	15 (2.1)
Household size (*N* = 1,282)				
	0–2 others	222 (17.3)	100 (17.7)	122 (17.0)
	3–4 others	557 (43.5)	207 (36.6)	350 (48.8)
	≥5 others	503 (39.2)	258 (45.7)	245 (34.2)
Persons per bedroom (*N* = 1,280)				
	0–2 persons	854 (66.7)	337 (59.8)	517 (72.2)
	>2–4 persons	387 (30.2)	206 (36.7)	181 (25.3)
	>4 persons	39 (3.0)	21 (3.7)	18 (2.5)
Child aged <5 years in household				
	No	821 (64.0)	345 (61.0)	476 (66.4)
	Yes	462 (36.0)	221 (39.0)	241 (33.6)
SARS-CoV-2 infection (*N* = 1,278)				
	Negative	1,134 (88.7)	469 (83.2)	665 (93.1)
	Positive	144 (11.3)	95 (16.8)	49 (6.9)

^*a*^ SARS-CoV-2, severe acute respiratory syndrome coronavirus 2, as detected by clinical transcription-mediated amplification testing from oropharyngeal specimens. We indicate the total number of individuals providing answers to each question with missing data.

^*b*^ We detail the characteristics of participants who reported speaking indigenous languages at home, versus other participants, in Table S11.

^*c*^ Variable collected among primary study participants only and not available for participants in the expanded study. We present disaggregated data for the primary and supplemental study populations in Table S1.

### Pneumococcal detection

Applying quantitative PCR to culture-enriched saliva samples, we detected the pneumococcal *lytA* gene at cycle threshold (c*_T_*) values below 40 in 342 participants (26.7% of 1,283; Table 2). Pneumococcal carriage (based on co-detection of *lytA* and *piaB* at c*_T_* <40) was present in 117 samples (9.1%), and higher-density pneumococcal carriage (c*_T_ <*35 for *lytA* and *piaB*) was present in 53 samples (4.1%). The prevalence of pneumococcal carriage was 11.1% (63/566) among participants recruited in clinical settings and 7.5% (54/717) among participants recruited through outreach testing. The prevalence of higher-density pneumococcal carriage was 5.5% (31/566) and 3.1% (22/717) in clinical and outreach venues, respectively, while the prevalence of any *lytA*-positive oral streptococcal carriage was 29.3% (166/566) and 24.5% (176/717) in clinical and outreach venues, respectively (Table S2). Restricting the sample to participants with negative SARS-CoV-2 testing results, the prevalence of pneumococcal carriage, higher-density pneumococcal carriage, and *lytA*-positive oral streptococcal carriage was 7.5% (85/1,134), 3.0% (34/1,134), and 24.6% (279/1,134), respectively, and did not differ appreciably according to the recruitment setting (<1% prevalence differences among participants enrolled in clinical and community settings).

**TABLE 2:** Detection of pneumococcal carriage in the study population^*a*^

Outcome	Participants, *n* (%)		
	All	Clinic	Outreach
	*N* = 1,283	*N* = 566	*N* = 717
Any pneumococcal carriage	117 (9.1)	63 (11.1)	54 (7.5)
Higher-density pneumococcal carriage	53 (4.1)	31 (5.5)	22 (3.1)
Any oral streptococcal (lytA-positive) carriage	342 (26.7)	166 (26.3)	176 (24.5)
Sample restricted to individuals with negative SARS-CoV-2 test results	*N* = 1,134	*N* = 469	*N* = 665
Any pneumococcal carriage	85 (7.5)	37 (7.9)	48 (7.2)
Higher-density pneumococcal carriage	34 (3.0)	15 (3.2)	19 (2.9)
Any oral streptococcal (lytA-positive) carriage	279 (24.6)	116 (24.7)	163 (24.5)

^*a*^ SARS-CoV-2, severe acute respiratory syndrome coronavirus 2, as detected by clinical transcription-mediated amplification testing from oropharyngeal specimens. Pneumococcal detection was defined as c_*T*_ values below 40 for both the *lytA* and *piaB* targets. Higher-density pneumococcal carriage was defined as c_*T*_ values below 35 for both the *lytA* and *piaB* targets. Oral streptococcal carriage detection was defined as c_*T*_ values below 40 for *lytA* target.

### Predictors of pneumococcal carriage

In comparison to non-carriers, pneumococcal carriers tended to more often be 30–39 years old and less often be 40–49 or ≥50 years old [odds ratio (OR) = 1.29 (95% confidence interval: 0.76–2.18), 0.77 (0.44–1.36), and 0.37 (0.18–0.75) for ages 30–39, 40–49, and ≥50 years, vs 18–29 years]. Carriers were more likely than non-carriers to speak indigenous languages in their households [OR = 3.94 (2.36–6.60)], less likely to have completed high school [OR = 0.48 (0.26–0.90)], and more likely to be engaged in field work tasks [OR = 2.02 (1.12–3.63)] (Tables 3 and 4). Pneumococcal carriers had also resided in the US for shorter periods of time than non-carriers [OR = 0.51 (0.27–0.96) for those living ≥18 years vs <5 years in the US], although this variable was inherently related to participant age. Speaking an indigenous language at home also predicted higher-density pneumococcal carriage [OR = 3.94 (2.36–6.60)], along with a lack of access to in-home laundry facilities [OR = 0.52 (0.28–0.94)].

**TABLE 3:** Characteristics of pneumococcal carriers and non-carriers^*a*^

Characteristics		Participants, *n* (%)					
		No pneumococcal carriage		Pneumococcal carriage		Higher-density pneumococcal carriage	
		Primary study population	Expanded study population	Primary study population	Expanded study population	Primary study population	Expanded study population
		*N* = 996	*N* = 1,166	*N* = 103	*N* = 117	*N* = 47	*N* = 53
Age							
	18–29	245 (24.6)	311 (26.7)	29 (28.2)	33 (28.2)	14 (29.8)	14 (26.4)
	30–39	235 (23.6)	266 (22.8)	37 (35.9)	40 (34.2)	20 (42.6)	22 (41.5)
	40–49	264 (26.5)	299 (25.6)	26 (25.2)	28 (23.9)	10 (21.3)	12 (22.6)
	≥50	252 (25.3)	290 (24.9)	11 (10.7)	16 (13.7)	3 (6.4)	5 (9.4)
Sex							
	Female	518 (52.0)	632 (54.2)	58 (56.3)	66 (56.4)	22 (46.8)	25 (47.2)
	Male	478 (48.0)	534 (45.8)	45 (43.7)	51 (43.6)	25 (53.2)	28 (52.8)
Country of birth							
	Mexico or other	869 (87.2)	960 (82.3)	90 (87.4)	97 (82.9)	41 (87.2)	44 (83.0)
	United States	127 (12.8)	206 (17.7)	13 (12.6)	20 (17.1)	6 (12.8)	9 (17.0)
Language spoken at home							
	English	50 (5.0)	106 (9.1)	6 (5.8)	12 (10.3)	4 (8.5)	6 (11.3)
	Spanish	866 (86.9)	979 (84)	68 (66)	76 (65)	28 (59.6)	32 (60.4)
	Indigenous	80 (8.0)	81 (6.9)	29 (28.2)	29 (24.8)	15 (31.9)	15 (28.3)
Annual household income (USD) (*N* = 1,212)							
	<25,000	498 (52.3)	566 (51)	53 (58.9)	56 (54.9)	19 (45.2)	21 (44.7)
	≥25,000	455 (47.7)	544 (49)	37 (41.1)	46 (45.1)	23 (54.8)	26 (55.3)
Smoking status (*N* = 1,098)							
	Never smoked	804 (80.8)	– –^*b*^	87 (84.5)	– –	38 (80.9)	– –
	Current or former smoker	191 (19.2)	– –	16 (15.5)	– –	9 (19.2)	– –
Years living in United States							
	<5 years	89 (10.3)	– –	16 (17.8)	– –	9 (22.0)	– –
	5–18 years	237 (27.3)	– –	28 (31.1)		15 (36.6)	
	≥18 years	542 (62.4)	– –	46 (51.1)	– –	17 (41.5)	– –
H-2A visa (*N* = 946)							
	No	798 (93.2)	– –	83 (92.2)	– –	37 (90.2)	– –
	Yes	58 (6.8)	– –	7 (7.8)	– –	4 (9.8)	– –
Educational attainment (*N* = 1,282)			– –				
	Less than high school	767 (77.1)	826 (70.9)	91 (88.3)	96 (82.1)	40 (85.1)	42 (79.3)
	High school diploma or higher	228 (22.9)	339 (29.1)	12 (11.7)	21 (17.9)	7 (14.9)	11 (20.7)
Child aged <5 years in household							
	No	633 (63.6)	753 (64.6)	58 (56.3)	68 (58.1)	21 (44.7)	25 (47.2)
	Yes	363 (36.4)	413 (35.4)	45 (43.7)	49 (41.9)	26 (55.3)	28 (52.8)
Household size (*N* = 1,282)							
	0–2 others	172 (17.3)	210 (18)	9 (8.7)	12 (10.3)	3 (6.4)	5 (9.4)
	3–4 others	424 (42.6)	503 (43.2)	46 (44.7)	54 (46.2)	20 (42.6)	23 (43.4)
	≥5 others	399 (40.1)	452 (38.8)	48 (46.6)	51 (43.6)	24 (51.1)	25 (47.2)
Persons per bedroom (*N* = 1,280)							
	0–2 persons	644 (64.7)	789 (67.7)	53 (52.5)	65 (56.5)	20 (43.5)	24 (46.2)
	>2–4 persons	322 (32.4)	345 (29.6)	40 (39.6)	42 (36.5)	21 (45.7)	23 (44.2)
	>4 persons	29 (2.9)	31 (2.7)	8 (7.9)	8 (7.0)	5 (10.9)	5 (9.6)
Washing machine in residence (*N* = 1,099)							
	No	364 (36.5)	– –	44 (42.7)	– –	26 (55.3)	– –
	Yes	632 (63.5)	– –	59 (57.3)	– –	21 (44.7)	– –
Field worker occupation (*N* = 1,089)							
	No	256 (25.9)	– –	14 (13.7)	– –	5 (10.6)	– –
	Yes	731 (74.1)	– –	88 (86.3)	– –	42 (89.4)	– –
Commuted to work with non-household members (*N* = 1,078)							
	No	645 (66.0)	– –	59 (58.4)	– –	27 (58.7)	– –
	Yes	332 (34.0)	– –	42 (41.6)	– –	19 (41.3)	– –
Commuted to work in employer-provided bus (*N* = 899)							
	No	737 (90.2)	– –	68 (82.9)	– –	32 (84.2)	– –
	Yes	80 (9.8)	– –	14 (17.1)	– –	6 (15.8)	– –
Number of people outside of household that you commute with (*N* = 912)							
	0 others	552 (66.7)	– –	48 (57.1)	– –	24 (60.0)	– –
	1–14 others	224 (27.1)	– –	26 (31.0)	– –	10 (25.0)	– –
	≥15 others	52 (6.3)	– –	10 (11.9)	– –	6 (15.0)	– –
Wearing a face covering when in proximity to others outside the home (*N* = 1,099)							
	Some of the time, rarely, or never	78 (7.8)	– –	8 (7.8)	78 (7.8)	5 (10.6)	– –
	All or most of the time	918 (92.2)	– –	95 (92.2)	918 (92.2)	42 (89.4)	– –

^*a*^ We indicate the total number of individuals providing answers to each question with missing data.

^*b*^ Dashes (– –) indicate data not available for the expanded study population.

**TABLE 4:** Associations of pneumococcal carriage and high-density carriage with participant characteristics^*a*^

Characteristic		Odds ratio (95% CI)			
		Any pneumococcal carriage		Higher-density pneumococcal carriage	
		Primary study population	Expanded study population	Primary study population	Expanded study population
Age					
	18–29	ref.	ref.	ref.	ref.
	30–39	1.29 (0.76–2.18)	1.38 (0.84–2.26)	1.46 (0.71–3.01)	1.79 (0.89–3.63)
	40–49	0.77 (0.44–1.36)	0.80 (0.47–1.37)	0.61 (0.26–1.41)	0.79 (0.35–1.76)
	≥50	0.37 (0.18–0.75)	0.53 (0.28–0.98)	0.21 (0.06–0.76)	0.40 (0.14–1.30)
Sex					
	Female	ref.	ref.	ref.	ref.
	Male	0.82 (0.54–1.24)	0.88 (0.60–1.30)	1.18 (0.65–2.14)	1.25 (0.71–2.20)
Country of birth					
	Mexico or other	ref.	ref.	ref.	ref.
	United States	1.10 (0.59–2.06)	1.13 (0.67–1.90)	1.21 (0.49–2.98)	1.23 (0.57–2.65)
Language spoken at home					
	English or Spanish	ref.	ref.	ref.	ref.
	Indigenous	3.94 (2.36–6.60)	3.80 (2.28–6.33)	4.18 (2.08–8.40)	4.03 (2.01–8.08)
Household income					
	Below $25,000	ref.	ref.	ref.	ref.
	$25,000 or above	0.78 (0.50–1.21)	0.89 (0.59–1.35)	1.37 (0.73–2.57)	1.39 (0.76–2.52)
Marital status					
	Not married or living as married	ref.	ref.	ref.	ref.
	Married or living as married	1.38 (0.88–2.14)	1.38 (0.92–2.07)	1.91 (0.97–3.75)	1.99 (1.06–3.72)
Cigarette smoking					
	Never smoked	ref.	– –	ref.	– –
	Current or former smoker	0.78 (0.44–1.37)	– –	1.04 (0.49–2.21)	– –
Years living in United States					
	<5 years	ref.	– –	ref.	– –
	5–18 years	0.68 (0.35–1.34)	– –	0.66 (0.27–1.60)	– –
	≥18 years	0.51 (0.27–0.96)	– –	0.36 (0.15–0.85)	– –
H-2A visa					
	No	ref.	– –	ref.	– –
	Yes	1.00 (0.43–2.33)	– –	1.30 (0.43–3.95)	– –
Educational attainment					
	Less than high school	ref.	ref.	ref.	ref.
	High school diploma or higher	0.48 (0.26, 0.90)	0.59 (0.36, 0.97)	0.68 (0.30, 1.56)	0.76 (0.38, 1.52)
Child aged <5 years in household					
	No	ref.	ref.	ref.	ref.
	Yes	1.45 (0.95–2.20)	1.38 (0.93–2.05)	2.42 (1.32–4.43)	2.25 (1.28–3.98)
Household size					
	0–2 others	ref.	ref.	ref.	ref.
	3–4 others	2.20 (1.04–4.64)	1.96 (1.02–3.78)	2.89 (0.84–9.97)	2.00 (0.74–5.40)
	≥5 others	2.41 (1.15–5.07)	1.98 (1.03–3.83)	3.60 (1.06–12.27)	2.32 (0.86–6.22)
Persons per bedroom					
	≤2 people	ref.	ref.	ref.	ref.
	>2 people to 4 people	1.48 (0.96–2.30)	1.42 (0.94–2.16)	2.00 (1.06–3.79)	2.05 (1.13–3.74)
	Over 4 people	2.84 (1.20–6.73)	2.55 (1.09–5.95)	4.81 (1.62–14.29)	4.38 (1.50–12.79)
Washing machine in residence					
	No	ref.	– –^*b*^	ref.	– –
	Yes	0.83 (0.55–1.27)	– –	0.52 (0.28–0.94)	– –
Field worker occupation					
	No	ref.	– –	ref.	– –
	Yes	2.02 (1.12–3.63)	– –	2.63 (1.02–6.77)	– –
Workplace setting					
	Indoors and outdoor	ref.	– –	ref.	– –
	Outdoors only	1.67 (0.96–2.92)	– –	1.46 (0.67–3.20)	– –
Commuted to work with non-household members					
	No	ref.	– –	ref.	– –
	Yes	1.35 (0.89–2.05)	– –	1.31 (0.72–2.40)	– –
Commuted to work in employer-provided bus					
	No	ref.	– –	ref.	– –
	Yes	1.96 (1.05–3.65)	– –	1.82 (0.74–4.50)	– –
Number of people outside of household that you commute with					
	0 others	ref.	– –	ref.	– –
	1–14 others	1.26 (0.76–2.09)	– –	0.95 (0.45–2.04)	– –
	≥15 others	2.36 (1.12–4.95)	– –	2.95 (1.14–7.61)	– –
Wearing a face covering when in proximity to others outside the home					
	All or most of the time	ref.	– –	ref.	– –
	Some of the time, rarely, or never	1.12 (0.52–2.41)	– –	1.68 (0.63–4.46)	– –

^*a*^ Odds ratios are computed via conditional logistic regression models stratified by recruitment venue and SARS-CoV-2 infection status.

^*b*^ Dashes (– –) indicate data not available for the expanded study population.

Pneumococcal carriers were more likely than non-carriers to commute to work on employer-provided buses [OR = 1.96 (1.05–3.65)] and tended to commute with persons from outside their household more often than non-carriers [OR = 1.26 (0.76–2.09) and 2.36 (1.12–4.95) for participants commuting with 1–14 and ≥15 others from outside their household, as compared to participants who did not commute with persons from outside their household].

With regard to household exposures, pneumococcal carriers were more likely than non-carriers to be exposed to a child aged <5 years at home [OR = 1.45 (0.95–2.20)]; tended more often to live in households with >2–4 persons per bedroom [OR = 1.48 (0.96–2.30) vs <2 persons per bedroom] or >4 persons per bedroom [OR = 2.84 (1.20–6.73) vs <2 persons per bedroom]; and tended to live in households with 3–4 others [OR = 2.20 (1.04–4.64) vs <3 others] or ≥5 others [OR = 2.41 (1.15–5.07) vs <3 others; Table 4]. For the same exposures, greater effect sizes were apparent for the outcome of higher-density pneumococcal carriage.

We next aimed to determine whether the relationship between household overcrowding and pneumococcal carriage depended upon the presence of children in the household (Table 5; Table S3). Among participants residing within households without a child aged <5 years, those living in households with >2 persons per bedroom had 2.05 (1.18–3.59)-fold greater odds of pneumococcal carriage and 5.01 (1.96–12.83)-fold greater odds of higher-density pneumococcal carriage in comparison to those living in households with ≤2 persons per bedroom. Likewise, those living in households with ≥3 other inhabitants had 2.13 (0.98–4.63)-fold greater odds of pneumococcal carriage and 3.25 (0.73–14.37)-fold greater odds of higher-density pneumococcal carriage in comparison to those living in households with <3 others.

**TABLE 5:** Associations of pneumococcal carriage and higher-density carriage with crowding among participants without residential exposure to children aged <5 years^*a*^

Characteristic		Odds ratio (95% CI)			
		Any pneumococcal carriage		Higher-density pneumococcal carriage	
		Primary study population	Expanded study population	Primary study population	Expanded study population
Household size					
	0–2 others	ref.	ref.	ref.	ref.
	3–4 others	2.10 (0.93–4.72)	1.95 (0.94–4.04)	2.93 (0.63–13.66)	2.22 (0.61–8.07)
	≥5 others	2.18 (0.91–5.23)	1.98 (0.89–4.41)	3.81 (0.78–18.64)	2.99 (0.78–11.51)
	≥3 others	2.13 (0.98–4.63)	1.96 (0.97–3.95)	3.25 (0.73–14.37)	2.48 (0.72–8.57)
Persons per bedroom					
	≤2 persons	ref.	ref.	ref.	ref.
	>2–4 persons	1.78 (0.98–3.23)	1.68 (0.95–2.97)	4.41 (1.67–11.83)	4.00 (1.64–9.74)
	≥4 persons	2.93 (0.86–9.90)	2.58 (0.77–8.59)	6.49 (1.15–36.63)	5.14 (0.94–28.00)
	>2 persons	2.05 (1.18–3.59)	1.92 (1.13–3.25)	5.01 (1.96–12.83)	4.42 (1.90–10.28)

^*a*^ Odds ratios are computed via conditional logistic regression models stratified by recruitment venue and SARS-CoV-2 infection status. Due to the limited statistical power within the subset of participants not exposed to children aged <5 years within their households, we present disaggregated estimates (per the analyses in Table 4) for three exposure categories as well as estimates for exposure to 0–2 vs ≥3 household members and exposure to households with ≤2 vs >2 persons per bedroom.

We also aimed to address the possibility that crowded housing and commuting exposures could be related to arrangements such as living in dormitory-style or employer-provided facilities. We repeated analyses of the association of commuting arrangements with pneumococcal carriage within the subgroup of participants who lived in households with ≤2 persons per bedroom. Within this subgroup, participants commuting to work in an employer-provided bus had 2.03 (0.66–6.27)-fold greater odds of pneumococcal carriage than those not commuting in an employer-provided bus, while those who commuted in vehicles with 1–14 and ≥15 others from outside their household had 1.22 (0.61–2.43)- and 1.71 (0.37–7.94)-fold greater odds of pneumococcal carriage than those who did not commute with persons from outside their household (Table 6; Table S4).

**TABLE 6:** Associations of pneumococcal carriage and higher-density carriage with commuting exposures among participants without exposure to household crowding^*a*^

Characteristic		Odds ratio (95% CI)	
		Any pneumococcal carriage	Higher-density pneumococcal carriage
Commuted to work in employer-provided bus (*N* = 899)			
	No	ref.	ref.
	Yes	2.03 (0.66–6.27)	1.07 (0.14–8.50)
Number of people outside of household that you commute with (*N* = 912)			
	0 others	ref.	ref.
	1–14 others	1.22 (0.61–2.43)	0.64 (0.20–2.01)
	≥15 others	1.71 (0.37–7.94)	1.66 (0.20–13.81)

^*a*^ Odds ratios are computed via conditional logistic regression models stratified by recruitment venue and SARS-CoV-2 infection status. Commuting exposures were not collected for the extended population; therefore, analysis is limited to the primary study population.

### Pneumococcal carriage density

**Fig 1:**
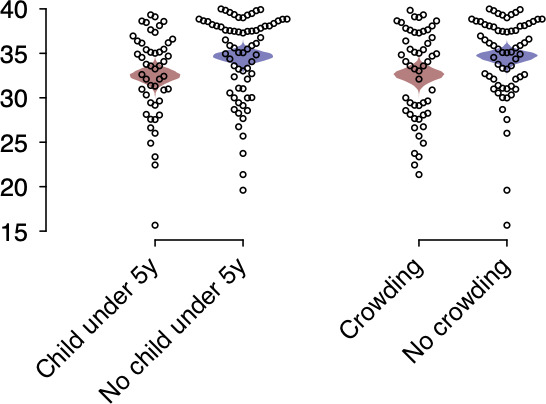
Factors predicting pneumococcal carriage density. We illustrate *piaB* c*_T_* value distributions among participants found to carry pneumococci based on their exposure to children aged <5 years in the household and crowding in the household. Crowding is defined as living in a household with ≥2 persons per bedroom. Mean between-group differences in c*_T_* values were −2.04 (−3.73, −0.36) for households with or without children aged <5 years and −2.44 (−4.11, −0.80) for crowded vs uncrowded households; including data from the expanded study population, mean between-group differences in c*_T_* values were −2.08 (−3.85, −0.33) for crowded vs uncrowded households and −2.17 (−3.95, −0.41) for households with or without children aged <5 years. Shaded polygons illustrate the distributions of mean estimates within each stratum based on bootstrap resampling.

Among participants carrying pneumococci, those living with a child aged <5 years, on average, had *piaB* c*_T_* values 2.04 (0.36–3.73) units lower than those not living with a child aged <5 years (Fig. 1; Tables S5 and S6). Similarly, pneumococcal carriers exposed to household overcrowding (defined as >2 persons per bedroom in the household), on average, had *piaB* c*_T_* values 2.44 (0.80–4.11) units lower than those not exposed to household overcrowding. We did not identify any participant characteristics or exposures associated with differences in *lytA* c*_T_* values.

### Serotype distribution

The most commonly detected serotypes among pneumococcal carriers were 15A (*n* = 22, 18.8%), 24F/A/B (*n* = 14, 12.0%), 10A (*n* = 12, 10.3%), and 23B (*n* = 10, 8.5%; Table 7). Among 78 participants with any serotype detected (66.7% of all 117 pneumococcal carriers), 13 (16.7%), 14 (17.9%), and 32 (41.0%) participants carried serotypes covered by PCV13, PCV15, and PCV20, respectively. The detection of a pneumococcal serotype was 33.2% (3.2%–67.9%) more likely among pneumococcal carriers with concurrent SARS-CoV-2 infection than among those without concurrent SARS-CoV-2 infection [26/32 (81.3%) and 52/85 (61.2%), respectively], in line with our prior finding of higher pneumococcal carriage density among participants with concurrent SARS-CoV-2 infection (26). Overall, serotype distributions did not differ appreciably among participants who were infected or not infected with SARS-CoV-2 (*P* > 0.1 by $\chi^{2}$ test).

**TABLE 7:** Pneumococcal serotypes detected, stratified by SARS-CoV-2 infection status^*a*^

		Participants, *n* (%)		
Serotype		All participants	SARS-CoV-2 negative participants	SARS-CoV-2 positive participants
		*N* = 117	*N* = 85	*N* = 32
15A		22 (18.8)	15 (17.6)	7 (21.9)
24F/A/B		14 (12)	8 (9.4)	6 (18.8)
10A		12 (10.3)	10 (11.8)	2 (6.3)
23B		10 (8.5)	7 (8.2)	3 (9.4)
22A/F		5 (4.3)	3 (3.5)	2 (6.3)
	22A	3 (2.6)	2 (2.4)	1 (3.1)
	22F	1 (0.9)	0 (0)	1 (3.1)
	Indistinguishable 22A/F	1 (0.9)	1 (1.2)	0 (0)
9L/N		5 (4.3)	2 (2.4)	3 (9.4)
35F/47F		5 (4.3)	5 (5.9)	0 (0)
3		4 (3.4)	1 (1.2)	3 (9.4)
34		4 (3.4)	2 (2.4)	2 (6.3)
13		4 (3.4)	4 (4.7)	0 (0)
28A/F		4 (3.4)	3 (3.5)	1 (3.1)
11A/D/E		4 (3.4)	2 (2.4)	2 (6.3)
14		3 (2.6)	2 (2.4)	1 (3.1)
15B/C		3 (2.6)	2 (2.4)	1 (3.1)
23F		2 (1.7)	2 (2.4)	0 (0)
7F/A		2 (1.7)	1 (1.2)	1 (3.1)
16F		1 (0.9)	1 (1.2)	0 (0)
19A		1 (0.9)	0 (0)	1 (3.1)
6A/B		1 (0.9)	1 (1.2)	0 (0)
12F/44		0 (0)	0 (0)	0 (0)
8		0 (0)	0 (0)	0 (0)
38		0 (0)	0 (0)	0 (0)
1		0 (0)	0 (0)	0 (0)
20		0 (0)	0 (0)	0 (0)
Any serotype detected		78 (66.7)	52 (61.2)	26 (81.3)
Serotypes grouped according to PCV inclusion				
	PCV13 serotypes	13 (11.1)	7 (8.2)	6 (18.8)
	PCV15 serotypes	14 (12.0)	7 (8.2)	7 (21.9)
	PCV20 (including 15B/C)	32 (27.4)	21 (24.7)	11 (34.4)
Number of serotypes detected per sample				
	1	53 (45.3)	34 (40.0)	19 (59.4)
	2	20 (17.1)	16 (18.8)	4 (12.5)
	3	3 (2.6)	1 (1.2)	2 (6.3)
	4	2 (1.7)	1 (1.2)	1 (3.1)

^*a*^ Serotype findings are presented only for specimens where serotype-specific c_*T*_ values were within the range of ±2 from the *piaB* c_*T*_ value. Serotypes 4, 9V/9A, 17F, 21, 23A, 33A/33F, and 35B are excluded due to the low specificity of these probes in previous publications. Serogroup 6A/B was differentiated from 6A/B/C/D using conventional PCR methods.

### Symptoms

Among participants not infected with SARS-CoV-2, the odds of reporting any solicited symptoms or any respiratory symptoms did not differ appreciably according to pneumococcal carriage detection or carriage density (Tables S7 and S8). However, higher-density pneumococcal carriers had greater odds of reporting fever [OR = 4.72 (1.27–17.45)], sweating [OR = 4.42 (1.21–16.14)], and difficulty breathing [OR = 9.16 (2.36–35.48)] in comparison to participants who did not concurrently carry pneumococci. Within analyses that included all participants (matched on SARS-CoV-2 infection status), pneumococcal carriers had greater odds of reporting pressure in the ears [OR = 3.49 (1.32–9.23)], fever [OR = 2.63 (1.28–5.41)], chills [OR = 2.04 (1.03–4.02)], difficulty breathing [OR = 3.12 (1.34–7.23)], and shortness of breath [OR = 3.72 (1.39–9.94)]. The same symptoms were associated with higher-density pneumococcal carriage (Tables S9 and S10).

## DISCUSSION

Our findings provide insight into several facets of pneumococcal carriage among adults within a population with low socioeconomic status and prevalent exposure to overcrowding. Within our study, 7.5% of participants without SARS-CoV-2 infection carried pneumococci, including 7.9% of those recruited in clinics and 7.2% of those recruited in community settings. Our findings implicate exposure to young children (aged <5 years) as a key risk factor for pneumococcal carriage, consistent with other studies enrolling adults (16, 27). However, we also found that exposure to household overcrowding (>2 persons per bedroom) was independently associated with an increased risk of pneumococcal carriage, including among adults in households without young children. Each of these associations was strengthened when evaluating an outcome of higher-density pneumococcal carriage (*lytA* and *piaB* c*_T_* < 35). Because higher-density carriage can be a transient state resulting from recent pneumococcal acquisition, these findings may indicate that individuals with residential exposure to young children and overcrowding acquire pneumococci with greater frequency. Taken together, our findings suggest that in addition to transmission from young children to adults, transmission among adults may be an important feature of pneumococcal epidemiology in populations with prevalent exposure to residential overcrowding. This finding from adult carriage data may help to explain observations such as the occurrence of pneumococcal disease outbreaks among otherwise healthy, non-elderly adults living or working in close, congregate settings (17–22).

Consistent with other indications of transmission among adults in crowded settings, pneumococcal carriage was also more common among participants who commuted to work in employer-provided buses or via other modes of transportation shared with ≥15 others from outside their household. Directionally similar findings persisted in analyses subset to participants who did not live in overcrowded households. Although underpowered due to the limited sample size, consistency of effect estimates within this subgroup suggests that the finding cannot owe exclusively to the fact that many individuals who commuted to work via such arrangements also lived in close, congregate settings such as employer-provided or dormitory-style housing. Crowded vehicles may, thus, represent a setting for pneumococcal transmission within our study population, who often rely on such means of transportation to reach remote work sites. This finding is consistent with meningococcal outbreak investigations implicating school buses as settings for transmission (28).

Our study also identified several risk factors for pneumococcal carriage related to lower socioeconomic status within this community, including indigenous ethnic or cultural background (indicated by speaking an indigenous language at home), lower educational attainment, and engagement in fieldwork tasks (which receive lower pay in comparison to other agricultural occupations such as machine operation or truck driving). Individuals without access to in-home laundry facilities were also at increased risk of higher-density carriage. This finding may reflect similar socioeconomic status considerations or may result from transmission within laundromats (*lavanderías*), which represent important community gathering places within the study setting and remained open during the pandemic.

Because our study was undertaken during the early phases of the COVID-19 pandemic, our questionnaires also addressed behaviors aiming to reduce pathogen transmission. Whereas the use of face coverings was not associated with participants’ risk of pneumococcal carriage, prior studies have suggested that face masks may reduce the risk of carriage acquisition among healthcare workers (29) and Hajj pilgrims (30). The effectiveness of this approach may depend, in part, upon the type of face coverings worn and the relative contributions of airborne, droplet, or fomite exposures to pneumococcal carriage acquisition. While associations of pneumococcal carriage with close-contact exposures such as crowded housing and commuting conditions in our study are more consistent with droplet than airborne routes of spread, the understanding of pneumococcal transmission mechanisms remains limited.

Overall, 11.1%, 12.0%, and 27.4% of pneumococcal carriers in our study were found to carry serotypes targeted by PCV13, PCV15, and PCV20, respectively. These findings are broadly in agreement with prior evidence (31) that PCV20 (in comparison to PCV15) offers a greater improvement in coverage of serotypes prevalent in mucosal carriage or infections of the upper respiratory tract over PCV13. Pediatric PCV coverage is not known within our specific study setting, although prior studies suggest that coverage may be high within similar populations. Coverage has been estimated at 92% among children residing in the US with parents born in Mexico (32) and 95% among children in Mexico (33). Because a low proportion of pneumococcal carriage was associated with PCV13-targeted serotypes within our sample, it is unlikely that there is a reservoir of vaccine-serotype pneumococcal circulation in the study communities associated with unvaccinated or undervaccinated children. As the US and Mexico first implemented PCV7 in 2000 and 2006, respectively, most adults within our sample were ineligible to have received PCVs as children.

Despite prevalent overcrowding and low socioeconomic status within our study population, our estimates of adult pneumococcal carriage prevalence are similar to others from studies using saliva-based sampling and molecular detection methods (13–16). Several factors may account for this finding. Most (64%) of the participants were not exposed to children aged <5 years at home. Although we did not test for respiratory viruses other than SARS-CoV-2, circulation of such viruses was heavily suppressed during the study period, including in California (34); it is, thus, unlikely that such testing would identify appreciable numbers of infections. As infection with these viruses is associated with increased pneumococcal carriage acquisition and carriage density (27, 35, 36), their absence may have contributed to lower pneumococcal carriage prevalence and density during the study period. Lastly, assessments of pneumococcal carriage were a secondary objective of our study, which primarily addressed SARS-CoV-2 infection among farm workers in clinical and community settings. While our findings reveal characteristics associated with pneumococcal carriage, our results should not be considered to provide generalizable measures of pneumococcal carriage prevalence among farm workers—some of whom may have avoided participation due to fear of SARS-CoV-2 testing and implications for employment, income, and visibility to government authorities.

Several further limitations of our study should be noted. First, due to the evaluation of pneumococcal carriage as a secondary endpoint, our study was not designed to support causal inference about pathways of pneumococcal transmission. Many variables, such as indigenous ethnicity, education, income, and fieldwork tasks, are collinear and may each imperfectly measure similar socioeconomic status characteristics rather than serve as independent predictors of carriage. Second, our design is cross-sectional and, thus, does not offer an opportunity to establish temporality between exposures and pneumococcal carriage acquisition. Longitudinal studies sampling household members and other close contacts are needed to elucidate transmission dynamics. Such studies may also provide insight into the temporal relationship between pneumococcal carriage acquisition and onset of respiratory symptoms, which our analysis could not address. Third, the living and working conditions of farmworkers are distinct from those of the general public; thus, associations may vary in differing communities. For example, exposures of interest such as overcrowding were prevalent within our study population. Studies enrolling more diverse participants (including individuals with higher socioeconomic status) may be positioned to identify greater effect sizes due to greater variation in exposure. Fourth, stronger associations of measured risk factors with higher-density pneumococcal carriage may relate to the greater specificity of this outcome in comparison to pneumococcal carriage detection at the *lytA* and *piaB* c*_T_* < 40 threshold. Fifth, the infeasibility of colony-based pneumococcal detection methods for the assessment of adult carriage limited our ability to characterize serotype distributions in this study population. While restricting serotyping results to samples with c*_T_* values within ±2 of values observed for *piaB* helped to maximize specificity, we could not study all serotypes due to the non-specific association of some *cps* genes with other colonizing bacteria. Sixth, reliance on saliva samples alone without other accompanying upper respiratory specimens may have limited our ability to identify all pneumococcal carriers. Other studies using similar molecular approaches have reported 85%–87% sensitivity of saliva-only sampling relative to the combined use of saliva and nasopharyngeal/oropharyngeal or trans-nasal and trans-oral specimens (37, 38); a lack of gold standard prohibits the determination of specificity, although the use of a two-probe (*lytA* and *piaB*) qPCR protocol limits the risk of false positive detections in our study (39). Last, our study was not designed or powered to support inferential hypothesis testing; point estimates and confidence intervals should be interpreted as measures of effect sizes rather than hypothesis tests. Subgroup analyses, in particular, are underpowered and are intended to evaluate consistency in the directional nature of relationships observed within the larger sample rather than to support hypothesis testing.

In conclusion, our study identifies exposure to young children and residential overcrowding—including in households without young children—as important risk factors for pneumococcal carriage among adults in low-income agricultural communities within California. The association of these factors with higher carriage density further suggests that individuals encountering young children or crowded conditions within the residential environment acquire pneumococcal carriage with greater frequency. While confirming prior understanding that young children serve as a reservoir for the transmission of pneumococci, these findings also suggest that transmission among adults may play an important feature in pneumococcal epidemiology among populations exposed to overcrowding. Strategies to prevent transmission under such conditions, or to reduce individuals’ risk of severe pneumococcal disease through vaccination, may be of value for certain populations.

## MATERIALS AND METHODS

### Study setting

The Salinas Valley in Monterey County, California, is home to an agricultural workforce of approximately 50,000 resident workers and 40,000 seasonal workers (24). Most are undocumented Latino migrants from Mexico and Central American countries, who often reside in crowded multigenerational housing, including makeshift residences (40, 41). The local workforce also includes holders of temporary agricultural H-2A visas, many of whom receive employer-provided group housing within motels or dormitories (42). We undertook the parent study in partnership with Clínica de Salud del Valle de Salinas (CSVS), a Federally Qualified Health Center focused on providing health care for agricultural workers and their families within the region. At the time of our study, CSVS offered SARS-CoV-2 testing at no cost to all members of the community, both at their network of Salinas Valley clinics and off-site at community testing events held at housing complexes, agricultural fields, community health fairs, and other sites.

Shelter-in-place recommendations and other non-pharmaceutical interventions were in place during the study period. We have previously described compliance with such guidance within the study population in home, workplace, and community settings (43). Overall, 92% of participants reported routinely wearing face coverings when outside of their household in close proximity to others, 10% reported attending social gatherings outside of their household, and 5% reported attending indoor social gatherings, which were prohibited throughout the study period. Roughly half of participants reported fever or symptom screening at their workplaces. School closures were in force throughout Monterey County. While guidance for the reopening of childcare facilities was tied to county-level COVID-19 case reporting, informal childcare arrangements may have remained in place to enable continued workforce participation for parents.

### Study design

Enrollment for the study occurred between 16 July and 30 November 2020. For the primary study, we invited non-pregnant farmworkers aged ≥18 years who spoke English or Spanish and who received SARS-CoV-2 testing via CSVS at clinical and non-clinical settings to participate in a study on risk factors for SARS-CoV-2 infection. To expand the study sample for analyses specifically focused on pneumococcal carriage and SARS-CoV-2 infection (26), we also invited adults of any occupational status, who were not pregnant, to complete an abbreviated version of the study procedures. Regardless of the version of the study into which they were enrolled, all participants provided an ~3 mL saliva sample at the site where they completed their SARS-CoV-2 testing with CSVS (whether in clinical or non-clinical settings) and responded to a questionnaire addressing sociodemographic characteristics, household, and workplace exposures and clinical symptoms. After collection, untreated saliva samples were frozen at –20°C immediately and then stored at –80°C until delivery to the laboratory for processing.

### Molecular detection

We used previously validated protocols (38) for the molecular detection of pneumococcal carriage in saliva samples after a culture-enrichment step in which saliva samples were incubated on trypticase soy agar supplemented with 7% sheep’s blood and 5 mg/L gentamicin (Supplementary Methods). After bacterial DNA isolation, we evaluated pneumococcal carriage based on the presence of *lytA* and *piaB* genes via qPCR. We defined adults with *lytA* and *piaB* c*_T_* values <40 as pneumococcal carriers; those with c*_T_* values <35 for both *lytA* and *piaB* were considered to carry pneumococci at higher density. Adults with *lytA* c*_T_* values <40 but *piaB* c*_T_* values ≥40 were considered probable carriers of other *lytA*-positive oral Streptococci (44).

We accounted for SARS-CoV-2 infection status within statistical analyses (as described below) to correct for a potential synergistic relationship between SARS-CoV-2 and pneumococci, as identified within the primary study (26). To identify SARS-CoV-2 infection, oropharyngeal samples (collected at the same visit as study-related saliva samples) were tested via the qualitative Aptima nucleic acid transcription-mediated amplification assay (Hologic, Marlborough, MA, US).

### Serotyping

We conducted molecular serotyping using DNA from bacterial culture-enriched samples using a previously validated quadruplex qPCR technique (45). To overcome the low specificity of serotyping results from colony-free detection methods (e.g., due to the presence of other bacteria encoding the serotype-specific *cps* genes), we included only results where serotype-specific c*_T_* values fell within a range of ±2 relative to the *piaB* c*_T_* value. We excluded detections of serotypes 4, 9V/9A, 17F, 21, 23A, 33A/33F, and 35B due to prior evidence of low assay specificity for these serotypes (24, 27).

### Statistical analyses

We compared the distribution of risk factors among individuals according to pneumococcal carriage status. Risk factors included age, sex, country of birth, language spoken, household income, marital status, cigarette smoking, years residing in the US, H-2A visa status, educational attainment, exposure to children at home, household size and overcrowding, access to in-home laundry facilities, engagement in field work, commuting to work with persons from outside participants’ household or via employer-provided buses, and use of face coverings when outside the home. We calculated ORs using conditional logistic regression, matching participants on the recruitment venue (clinical or outreach testing site) and concurrent SARS-CoV-2 infection (positive or negative) via regression strata. Individuals without pneumococcal carriage provided the reference group for analyses of pneumococcal carriage or higher-density pneumococcal carriage outcomes.

We repeated the analyses within subgroups to verify effect size estimates for several exposures where collinearity was expected to occur. Because households with greater numbers of residents have a greater chance of including at least one child aged <5 years, we separately computed ORs for the association of pneumococcal carriage with household size and household overcrowding within the stratum of participants not exposed to children aged <5 years at home. Similarly, living in employer-provided or dormitory-style housing could account for both overcrowding and commuting arrangements. We, thus, also computed ORs for the association of pneumococcal carriage with commuting variables within the stratum of participants living in households with <2 persons per bedroom, who were unlikely to be living in employer-provided housing or other dormitory-style arrangements.

Because higher carriage density may indicate recent acquisition of pneumococcal carriage, we also explored associations of the risk factors listed above with carriage density to better understand potential sources of transmission. We used linear regression models to measure adjusted associations of each exposure with c*_T_* values for *piaB* among individuals found to carry pneumococcus; we used *piaB* as the primary target for measuring carriage density due to greater specificity as an indicator of pneumococcal carriage. The resulting effect measure can be interpreted as the adjusted mean difference in c*_T_* values when comparing pneumococcal carriers across exposure categories. We also report findings with respect to c*_T_* values for *lytA* as a supplemental analysis, noting that this measure may relate to the density of other *lytA*-positive oral Streptococci, even among pneumococcal carriers, due to its lower specificity.

Last, we aimed to assess whether pneumococcal carriage was related to the presence of concurrent respiratory symptoms. We stratified the analysis among the entire study population and among individuals with negative SARS-CoV-2 testing results; potential associations between symptoms and pneumococcal interactions with SARS-CoV-2 infection were addressed in prior analyses (26). We defined individuals experiencing respiratory symptoms as those who reported non-productive cough, productive cough, blocked nose, runny nose, sneezing, hoarseness, a tickling sensation in the throat, sore throat, painful sinuses, difficulty breathing, wheezing, or shortness of breath. We used conditional logistic regression models, stratified on recruitment clinical or outreach recruitment venue, to calculate ORs for outcomes of pneumococcal carriage and higher-density pneumococcal carriage. We also used linear regression to evaluate the mean difference in *lytA* and *piaB* c*_T_* values among pneumococcal carriers who experienced or did not experience symptoms, consistent with the density analyses described above.

For all analyses, we present ORs with accompanying 95% confidence intervals to describe effect sizes.

We conducted analyses using R (version 4.1.1; R Foundation for Statistical Computing, Vienna, Austria). Data for replication of the analyses are available via GitHub (46).

## SUPPLEMENTAL MATERIAL

Supplemental material spectrum.00879-24-s0001.pdf Supplemental methods; Tables S1 to S11.

ASM does not own the copyrights to Supplemental Material that may be linked to, or accessed through, an article. The authors have granted ASM a non-exclusive, world-wide license to publish the Supplemental Material files. Please contact the corresponding author directly for reuse.

## References

[1] Zarabi N, Aldvén M, Sjölander S, Fues Wahl H, Bencina G, Johnson KD, Silfverdal S-A. 2023. Clinical and economic burden of pneumococcal disease among adults in Sweden: a population-based register study. PLoS One 18:e0287581. doi:10.1371/journal.pone.0287581 37418396 PMC10328229

[2] Vos T, Lim SS, Abbafati C, Abbas KM, Abbasi M, Abbasifard M, Abbasi-Kangevari M, Abbastabar H, Abd-Allah F, Abdelalim A, et al.. 2020. Global burden of 369 diseases and injuries in 204 countries and territories, 1990–2019: a systematic analysis for the global burden of disease study 2019. The Lancet 396:1204–1222. doi:10.1016/S0140-6736(20)30925-9 PMC7567026 33069326

[3] Bogaert D, De Groot R, Hermans PWM. 2004. Streptococcus pneumoniae colonisation: the key to pneumococcal disease. Lancet Infect Dis 4:144–154. doi:10.1016/S1473-3099(04)00938-7 14998500

[4] Desai AP, Sharma D, Crispell EK, Baughman W, Thomas S, Tunali A, Sherwood L, Zmitrovich A, Jerris R, Satola SW, Beall B, Moore MR, Jain S, Farley MM. 2015. Decline in pneumococcal nasopharyngeal carriage of vaccine serotypes after the introduction of the 13-valent pneumococcal conjugate vaccine in children in Atlanta, Georgia. Pediatr Infect Dis J 34:1168–1174. doi:10.1097/INF.0000000000000849 26226445

[5] Kaur R, Pichichero M. 2023. Colonization, density, and antibiotic resistance of Streptococcus pneumoniae, Haemophilus influenzae, and Moraxella catarrhalis among PCV13-vaccinated infants in the first six months of life in Rochester, New York: a cohort study. J Pediatr Infect Dis Soc 12:135–142. doi:10.1093/jpids/piad004 36645216

[6] Yildirim I, Little BA, Finkelstein J, Lee G, Hanage WP, Shea K, Pelton SI, T, he Massachusetts Dept. of Public Health. 2017. Surveillance of pneumococcal colonization and invasive pneumococcal disease reveals shift in prevalent carriage serotypes in Massachusetts’ children to relatively low invasiveness. Vaccine 35:4002–4009. doi:10.1016/j.vaccine.2017.05.077 28645717

[7] Tvedskov ESF, Hovmand N, Benfield T, Tinggaard M. 2022. Pneumococcal carriage among children in low and lower-middle-income countries: a systematic review. Int J Infect Dis 115:1–7. doi:10.1016/j.ijid.2021.11.021 34800691

[8] Fortuna LBDP, Miranda FM, Antunes IMF, Silva AB, Cabral AS, Dolores ÍM, Cardoso-Marques NT, Teixeira LM, Neves FPG. 2023. Prevalence, capsular types, antimicrobial resistance and risk factors associated with pneumococcal carriage among children after long-term 10-valent pneumococcal conjugate vaccine use in Brazil. Vaccine 41:3111–3118. doi:10.1016/j.vaccine.2023.04.023 37061371

[9] Hill PC, Akisanya A, Sankareh K, Cheung YB, Saaka M, Lahai G, Greenwood BM, Adegbola RA. 2006. Nasopharyngeal carriage of Streptococcus pneumoniae in Gambian villagers. Clin Infect Dis 43:673–679. doi:10.1086/506941 16912937

[10] Huang SS, Johnson KM, Ray GT, Wroe P, Lieu TA, Moore MR, Zell ER, Linder JA, Grijalva CG, Metlay JP, Finkelstein JA. 2011. Healthcare utilization and cost of pneumococcal disease in the United States. Vaccine 29:3398–3412. doi:10.1016/j.vaccine.2011.02.088 21397721

[11] Wroe PC, Finkelstein JA, Ray GT, Linder JA, Johnson KM, Rifas-Shiman S, Moore MR, Huang SS. 2012. Aging population and future burden of pneumococcal pneumonia in the United States. J Infect Dis 205:1589–1592. doi:10.1093/infdis/jis240 22448012

[12] Melegaro A, Gay NJ, Medley GF. 2004. Estimating the transmission parameters of pneumococcal carriage in households. Epidemiol Infect 132:433–441. doi:10.1017/s0950268804001980 15188713 PMC2870123

[13] Arguedas A, Trzciński K, O’Brien KL, Ferreira DM, Wyllie AL, Weinberger D, Danon L, Pelton SI, Azzari C, Hammitt LL, Sá-Leão R, Brandileone M-CC, Saha S, Suaya J, Isturiz R, Jodar L, Gessner BD. 2020. Upper respiratory tract colonization with Streptococcus pneumoniae in adults. Expert Review of Vaccines 19:353–366. doi:10.1080/14760584.2020.1750378 32237926

[14] Mitsi E, Reiné J, Urban BC, Solórzano C, Nikolaou E, Hyder-Wright AD, Pojar S, Howard A, Hitchins L, Glynn S, et al.. 2022. Streptococcus pneumoniae colonization associates with impaired adaptive immune responses against SARS-CoV-2. J Clin Invest 132:e157124. doi:10.1172/JCI157124 35139037 PMC8970672

[15] Almeida ST, Paulo AC, Froes F, de Lencastre H, Sá-Leão R. 2021. Dynamics of pneumococcal carriage in adults: a new look at an old paradigm. J Infect Dis 223:1590–1600. doi:10.1093/infdis/jiaa558 32877517

[16] Wyllie AL, Mbodj S, Thammavongsa DA, Hislop MS, Yolda-Carr D, Waghela P, Nakahata M, Stahlfeld AE, Vega NJ, York A, Allicock OM, Wilkins G, Ouyang A, Siqueiros L, Strong Y, Anastasio K, Alexander-Parrish R, Arguedas A, Gessner BD, Weinberger DM. 2023. Persistence of pneumococcal carriage among older adults in the community despite COVID-19 mitigation measures. Microbiol Spectr 11:e0487922. doi:10.1128/spectrum.04879-22 37036377 PMC10269788

[17] Smit P, Oberholzer D, Hayden-Smith S, Koornhof HJ, Hilleman MR. 1977. Protective efficacy of pneumococcal polysaccharide vaccines. JAMA 238:2613–2616. 21973

[18] Banerjee A, Kalghatgi AT, Saiprasad GS, Nagendra A, Panda BN, Dham SK, Mahen A, Menon KD, Khan MA. 2005. Outbreak of pneumococcal pneumonia among military recruits. Med J Armed Forces India 61:16–21. doi:10.1016/S0377-1237(05)80111-X 27407697 PMC4923351

[19] Linkevicius M, Cristea V, Siira L, Mäkelä H, Toropainen M, Pitkäpaasi M, Dub T, Nohynek H, Puumalainen T, Rintala E, Laaksonen ME, Feuth T, Grönroos JO, Peltoniemi J, Frilander H, Lindström I, Sane J. 2019. Outbreak of invasive pneumococcal disease among shipyard workers, Turku, Finland, May to November 2019. Euro Surveill 24:1900681. doi:10.2807/1560-7917.ES.2019.24.49.1900681 31822326 PMC6905297

[20] Sanchez GV, Bourne CL, Davidson SL, Ellis M, Feldstein LR, Fay K, Brown NE, Geeter EF, Foster LL, Gilmore C, McIntyre MG, Taylor B, Velusamy S, Chochua S, Matanock AM. 2021. Pneumococcal disease outbreak at a state prison, Alabama, USA, September 1–October 10, 20181. Emerg Infect Dis 27:1949–1952. doi:10.3201/eid2707.203678 34152958 PMC8237874

[21] van Zandvoort K, Checchi F, Diggle E, Eggo RM, Gadroen K, Mulholland K, McGowan CR, le Polain de Waroux O, Rao VB, Satzke C, Flasche S. 2019. Pneumococcal conjugate vaccine use during humanitarian crises. Vaccine 37:6787–6792. doi:10.1016/j.vaccine.2019.09.038 31562004

[22] Mercat A, Nguyen J, Dautzenberg B. 1991. An outbreak of pneumococcal pneumonia in two men’s shelters. Chest 99:147–151. doi:10.1378/chest.99.1.147 1984946

[23] Ihekweazu C, Basarab M, Wilson D, Oliver I, Dance D, George R, Pebody R. 2010. Outbreaks of serious pneumococcal disease in closed settings in the post-antibiotic era: a systematic review. J Infect 61:21–27. doi:10.1016/j.jinf.2010.03.032 20381524

[24] Quandt SA, Brooke C, Fagan K, Howe A, Thornburg TK, McCurdy SA. 2015. Farmworker housing in the United States and its impact on health. New Solut 25:263–286. doi:10.1177/1048291115601053 26320122

[25] Lewnard JA, Mora AM, Nkwocha O, Kogut K, Rauch SA, Morga N, Hernandez S, Wong MP, Huen K, Andrejko K, Jewell NP, Parra KL, Holland N, Harris E, Cuevas M, Eskenazi B, CHAMACOS-Project-19 Study Team. 2021. Prevalence and clinical profile of severe acute respiratory syndrome coronavirus 2 infection among farmworkers, California, USA, June–November 2020. Emerg Infect Dis 27:1330–1342. doi:10.3201/eid2705.204949 33657340 PMC8084509

[26] Parker AM, Jackson N, Awasthi S, Kim H, Alwan T, Wyllie AL, Baldwin AB, Brennick NB, Moehle EA, Giannikopoulos P, Kogut K, Holland N, Mora-Wyrobek A, Eskenazi B, Riley LW, Lewnard JA. 2023. Association of upper respiratory Streptococcus pneumoniae colonization with severe acute respiratory syndrome coronavirus 2 infection among adults. Clin Infect Dis 76:1209–1217. doi:10.1093/cid/ciac907 36401872

[27] Harrison LH, Armstrong CW, Jenkins SR, Harmon MW, Ajello GW, Miller GB, Broome CV. 1991. A cluster of meningococcal disease on a school bus following epidemic influenza. Arch Intern Med 151:1005–1009. 2025124

[28] MacIntyre CR, Wang Q, Rahman B, Seale H, Ridda I, Gao Z, Yang P, Shi W, Pang X, Zhang Y, Moa A, Dwyer DE. 2014. Efficacy of face masks and respirators in preventing upper respiratory tract bacterial colonization and co-infection in hospital healthcare workers. Prev Med 62:1–7. doi:10.1016/j.ypmed.2014.01.015 24472436 PMC7172205

[29] Hoang V-T, Meftah M, Anh Ly TD, Drali T, Yezli S, Alotaibi B, Raoult D, Parola P, Pommier de Santi V, Gautret P. 2019. Bacterial respiratory carriage in French Hajj pilgrims and the effect of pneumococcal vaccine and other individual preventive measures: a prospective cohort survey. Travel Med Infect Dis 31:101343. doi:10.1016/j.tmaid.2018.10.021 30415081 PMC7110955

[30] Wolf E, Rowhani-Rahbar A, Tasslimi A, Matheson J, DeBolt C. 2016. Parental country of birth and childhood vaccination uptake in Washington state. Pediatrics 138:e20154544. doi:10.1542/peds.2015-4544 27358475 PMC4925079

[31] King LM, Andrejko KL, Kabbani S, Tartof SY, Hicks LA, Cohen AL, Kobayashi M, Lewnard JA. 2024. Outpatient visits and antibiotic use due to higher-valence pneumococcal vaccine serotypes. J Infect Dis:jiae142. doi:10.1093/infdis/jiae142 38498565 PMC11481348

[32] Gutierrez JP, Johri M. 2023. Socioeconomic and geographic inequities in vaccination among children 12 to 59 months in Mexico, 2012 to 2021. Rev Panam Salud Publica 47:e35. doi:10.26633/RPSP.2023.35 36751676 PMC9899057

[33] Mitsi E, Nikolaou E, Goncalves A, Blizard A, Hill H, Farrar M, et al.. 2024 RSV and rhinovirus asymptomatic upper airway infection increases pneumococcal carriage acquisition rates and density in adults whereas nasal inflammation is associated with bacterial shedding medRxiv. doi:10.1101/2024.02.08.24302534 39181126

[34] Cooksey GLS, Morales C, Linde L, Schildhauer S, Guevara H, Chan E, Gibb K, Wong J, Lin W, Bonin BJ, et al.. 2022. Severe acute respiratory syndrome coronavirus 2 and respiratory virus sentinel surveillance, California, USA, may 10, 2020–June 12, 2021. Emerg Infect Dis 28:9–19. doi:10.3201/eid2801.211682 34932449 PMC8714231

[35] Karppinen S, Teräsjärvi J, Auranen K, Schuez-Havupalo L, Siira L, He Q, Waris M, Peltola V. 2017. Acquisition and transmission of Streptococcus pneumoniae are facilitated during rhinovirus infection in families with children. Am J Respir Crit Care Med 196:1172–1180. doi:10.1164/rccm.201702-0357OC 28489454

[36] Jochems SP, Marcon F, Carniel BF, Holloway M, Mitsi E, Smith E, Gritzfeld JF, Solórzano C, Reiné J, Pojar S, Nikolaou E, German EL, Hyder-Wright A, Hill H, Hales C, de Steenhuijsen Piters WAA, Bogaert D, Adler H, Zaidi S, Connor V, Gordon SB, Rylance J, Nakaya HI, Ferreira DM. 2018. Inflammation induced by influenza virus impairs human innate immune control of pneumococcus. Nat Immunol 19:1299–1308. doi:10.1038/s41590-018-0231-y 30374129 PMC6241853

[37] Krone CL, Wyllie AL, van Beek J, Rots NY, Oja AE, Chu MLJN, Bruin JP, Bogaert D, Sanders EAM, Trzciński K. 2015. Carriage of Streptococcus pneumoniae in aged adults with influenza-like illness. PLOS ONE 10:e0119875. doi:10.1371/journal.pone.0119875 25789854 PMC4366201

[38] Wyllie AL, Rümke LW, Arp K, Bosch AATM, Bruin JP, Rots NY, Wijmenga-Monsuur AJ, Sanders EAM, Trzciński K. 2016. Molecular surveillance on Streptococcus pneumoniae carriage in non-elderly adults: little evidence for pneumococcal circulation independent from the reservoir in children. Sci Rep 6:34888. doi:10.1038/srep34888 27713565 PMC5054371

[39] Miellet WR, Almeida ST, Trzciński K, Sá-Leão R. 2023. Streptococcus pneumoniae carriage studies in adults: importance, challenges, and key issues to consider when using quantitative PCR-based approaches. Front. Microbiol 14:1122276. doi:10.3389/fmicb.2023.1122276 36910231 PMC9994646

[40] Bradman A, Whitaker D, Quirós L, Castorina R, Henn BC, Nishioka M, Morgan J, Barr DB, Harnly M, Brisbin JA, Sheldon LS, Mckone TE, Eskenazi B. 2007. Pesticides and their metabolites in the homes and urine of farmworker children living in the Salinas valley, CA. J Expo Sci Environ Epidemiol 17:331–349. doi:10.1038/sj.jes.7500507 16736054

[41] Bradman A, Chevrier J, Tager I, Lipsett M, Sedgwick J, Macher J, Vargas AB, Cabrera EB, Camacho JM, Weldon R, Kogut K, Jewell NP, Eskenazi B. 2005. Association of housing disrepair indicators with cockroach and rodent infestations in a cohort of pregnant Latina women and their children. Environ Health Perspect 113:1795–1801. doi:10.1289/ehp.7588 16330367 PMC1314924

[42] Aguilar-Gaxiola SA, Ramirez SM, Kissam E. 2021. A reality check from the fields—what’s next?. JAMA Netw Open 4:e2125128. doi:10.1001/jamanetworkopen.2021.25128 34524443

[43] Mora AM, Lewnard JA, Kogut K, Rauch SA, Hernandez S, Wong MP, Huen K, Chang C, Jewell NP, Holland N, Harris E, Cuevas M, Eskenazi B, CHAMACOS-Project-19 Study Team. 2021. Risk factors associated with SARS-CoV-2 infection among farmworkers in Monterey County, California. JAMA Netw Open 4:e2124116. doi:10.1001/jamanetworkopen.2021.24116 34524438 PMC8444020

[44] Hislop MS, Allicock OM, Thammavongsa DA, Mbodj S, Nelson A, Shaw AC, Weinberger DM, Wyllie AL. 2023. High levels of detection of nonpneumococcal species of Streptococcus in saliva from adults in the United States. Microbiol Spectr 11:e05207–22. doi:10.1128/spectrum.05207-22 37067447 PMC10269540

[45] Velusamy S, Tran T, Mongkolrattanothai T, Walker H, McGee L, Beall B. 2020. Expanded sequential quadriplex real-time polymerase chain reaction (PCR) for identifying pneumococcal serotypes, penicillin susceptibility, and resistance markers. Diagn Microbiol Infect Dis 97:115037. doi:10.1016/j.diagmicrobio.2020.115037 32265073

[46] Lewnard JA. 2024. Data and code repository for Streptococcus pneumoniae carriage study in farmworkers. https://github.com/joelewnard/sp-fw.

