# Supplementary material for: Upper respiratory Streptococcus pneumoniae colonization among working-age adults with prevalent exposure to overcrowding
Source: Microbiol Spectr. 2024 Jul 16;12(8):e00879-24. doi: 10.1128/spectrum.00879-24 (PMC11302326; doi:10.1128/spectrum.00879-24)
Supplement: Supplemental material — Supplemental methods; Tables S1 to S11. [file spectrum.00879-24-s0001.pdf]

Supporting information

Title: Predictors of upper respiratory *Streptococcus pneumoniae* colonization among working-age adults in California's Salinas Valley

Authors: Anna M. Parker[1], Nicole Jackson[2], Shevya Awasthi[2], Hanna Kim[2], Tess Alwan[2], Anne L. Wyllie[3], Katherine Kogut[4], Nina Holland[4], Ana M. Mora[4], Brenda Eskenazi[4], Lee W. Riley[2], Joseph A. Lewnard[1,2,5]

1. Division of Infectious Diseases & Vaccinology, School of Public Health, University of California, Berkeley, Berkeley, California 94720, United States
2. Department of Epidemiology of Microbial Diseases, Yale School of Public Health, New Haven, Connecticut 06510, United States
3. Center for Environmental Research & Community Health, School of Public Health, University of California, Berkeley, Berkeley, California 94720, United States
4. Division of Epidemiology, School of Public Health, University of California, Berkeley, Berkeley, California 94720, United States
5. Center for Computational Biology, College of Engineering, University of California, Berkeley, Berkeley, California 94720, United States

Contents of this supplement

Item	Title	Page
Text S1	Supplemental laboratory methods	2
Table S1	Characteristics of the primary and expanded study populations.	3
Table S2	Detection of other *lytA*-positive oral Streptococcal carriage within the primary and expanded study populations.	4
Table S3	Detection of pneumococcal carriage among participants exposed to varying levels of household crowding, stratified according to the presence a child aged <5 years in the household.	6
Table S4	Detection of pneumococcal carriage among participants exposed to various commuting environments, stratified according to the presence of household crowding.	7
Table S5	Associations of participant characteristics with detection of other *lytA*-positive oral Streptococcal carriage within the primary and expanded study populations.	8
Table S6	Association of participant characteristics with *lytA* and *piaB* c_T values among pneumococcal carriers.	10
Table S7	Associations of pneumococcal carriage with symptoms in last 2 weeks among study participants without SARS-CoV-2 infection.	11
Table S8	Association of symptoms with *lytA* and *piaB* c_T values among study participants without SARS-CoV-2 infection.	15
Table S9	Associations of pneumococcal carriage with symptoms in last 2 weeks among all study participants.	13
Table S10	Association of symptoms with *lytA* and *piaB* c_T values, among all study participants.	16

Supplemental laboratory methods

Sample processing and culture enrichment: Participants were instructed to provide the saliva that naturally pools in the mouth. These saliva specimens were frozen at –20ºC and then stored at –80ºC until transport to laboratory. Samples were thawed on ice after arrival at the laboratory; after thawing, 100µL of unprocessed saliva was spread onto trypticase soy agar supplemented with 7% sheep's blood and 5mg/L gentamicin. Plates were then incubated at 37ºC with 5% CO2 for 14-19 hours. Bacterial growth was harvested into 2.1mL of 10% glycerol brain-heart infusion broth (BHI), and stored at –20ºC. The remaining raw saliva not used for culture enrichment was stored at –80ºC.

Isolation of bacterial DNA: Culture-enriched samples were thawed and vortexed then transferred into sterile 1.5mL Eppendorf tubes prefilled with lysis buffer containing 40mg/mL lysozyme and 75U/mol mutanolysin. Next, the mixture was incubated for 90 minutes at 37ºC, followed by the addition of 20µL of Proteinase K and another incubation of 60 minutes at 56 ºC. Subsequent extraction steps used the QIAGEN DNeasy Blood and Tissue Kit (QIAGEN; Hilden, Germany) following manufacturer's instructions. DNA was eluted into 50µL elution buffer and nucleic acid concentration was measured using a NanoDrop spectrophotometer. Samples with a DNA concentration <20ng/µL underwent a second extraction. Experimental control DNA was extracted with the biolate method from pure culture. Briefly, colonies grown overnight on a TSA-GENT plate at 37ºC with 5% CO2 were extracted and suspended in 60µL nuclease-free water. The sample was then placed into a water bath heated to 100ºC and boiled for 10 minutes before being centrifuged for 10 minutes at 14,000 rpm. The supernatant was removed, and nucleic acid concentration was measured. Positive and negative control DNA was diluted with nuclease-free water to a concentration of 50ng/µL. All extracted DNA samples were stored at –20ºC.

Molecular detection of *S. pneumoniae:* Culture-enriched DNA was tested via quantitative PCR (qPCR) for presence of the *lytA* and *piaB* genes to determine pneumococcal presence (defined as identification with 40 or fewer cycle threshold values [c_T] for both genes). Gene detection via qPCR was conducted with primer concentrations at 400nM and 300nM, and probe concentrations at 75nM and 200nM, for lytA and piaB, respectively. All qPCR assays tested 2.5µL of DNA template in 25µL reactions. Thermal cycling conditions were as follows: 3 minutes at 95ºC for initial denaturation; 15 seconds at 98ºC for denaturation; and 30 seconds at 60ºC for annealing. Positive controls consisted of a diluted series of reference strain ATCC 49619. We used DNA from human saliva previously characterized as negative for *S. pneumoniae* and DNA from *E. coli* reference strain ATCC 25922 as our negative controls.

Table S1. Characteristics of the primary and expanded study populations.

Characteristics		Participants, *n* (%)		
		All	*Primary population*	*Supplemental population*
		N=1,283	*N*=1,099	*N*=184
Recruitment venue				
	Clinical setting	566 (44)	551 (50)	15 (8)
	Outreach setting	717 (56)	548 (50)	169 (92)
Age range, years				
	18-29	344 (27)	274 (25)	70 (38)
	30-39	306 (24)	272 (25)	34 (18)
	40-49	327 (25)	290 (26)	37 (20)
	≥50	306 (24)	263 (24)	43 (23)
Sex				
	Female	698 (54)	576 (52)	122 (66)
	Male	585 (46)	523 (48)	62 (34)
Country of birth				
	Mexico	1,012 (79)	917 (83)	86 (47)
	United States	226 (18)	140 (13)	95 (52)
	Other	45 (3.5)	42 (3.8)	3 (2)
Language spoken at home				
	Spanish	1,055 (82)	934 (85)	62 (34)
	English	118 (9)	56 (5)	121 (66)
	Indigenous language	110 (89)	109 (10)	1 (<1)
Education				
	Never attended school	63 (5)	62 (6)	1 (<1)
	Some primary school	460 (36)	422 (38)	38 (21)
	Primary school completed	251 (20)	235 (21)	16 (9)
	Some high school	148 (12)	139 (13)	9 (5)
	High school completed	360 (28)	240 (22)	120 (65)
Annual household income, USD (*N*=1,212)				
	<25,000	622 (51)	551 (53)	71 (42)
	25,000-34,999	284 (23)	257 (25)	27 (16)
	35,000-49,000	195 (16)	159 (15)	36 (21)
	≥ 50,000	111 (9)	76 (7)	35 (21)
Housing type				
	House	597 (47)	520 (47)	77 (42)
	Apartment	576 (45)	474 (43)	102 (55)
	Trailer/mobile home	45 (4)	43 (4)	2 (1)
	Hotel/motel	38 (3)	37 (3)	1 (<1)
	Other	27 (2)	25 (2)	2 (1)
Community of residence				
	Greenfield	309 (24)	309 (28)	0 (0)
	Salinas	485 (38)	485 (44)	0 (0)
	Other	489 (38)	305 (28)	184 (100)
Pneumococcal detection				
	lytA c_T <40	342 (27)	302 (27)	40 (22)
	lytA c_T <40 and *piaB* c_T <40	118 (9)	104 (10)	14 (18)
	lytA c_T <35 and *piaB* c_T <35	59 (5)	51 (5)	8 (4)

Table S2: Detection of other *lytA*-positive oral Streptococcal carriage within the primary and expanded study populations.

Characteristics		Participants, *n* (%)			
		No oral Streptococcal carriage detected		*Any oral Streptococcal carriage detected*	
		Primary study population	Expanded study population	Primary study population	Expanded study population
		N=797	N=941	N=302	N=342
Age					
	Mean, years (interquartile range)	40 (29-50)	39 (29-50)	40 (30-48)	39 (29-48)
Sex					
	Female	413 (51.8)	508 (54.0)	163 (54.0)	190 (55.6)
	Male	384 (48.2)	433 (46.0)	139 (46.0)	152 (44.4)
Country of birth					
	United States	106 (13.3)	170 (18.1)	34 (11.3)	56 (16.4)
	Mexico or other	691 (86.7)	771 (81.9)	268 (88.7)	286 (83.6)
Language spoken at home					
	English	42 (5.3)	88 (9.4)	14 (4.6)	30 (8.8)
	Spanish	698 (87.6)	795 (84.5)	236 (78.2)	260 (76.0)
	Indigenous	57 (7.2)	58 (6.0)	52 (17.2)	52 (15.2)
Annual household income					
	<25,000	406 (53.0)	464 (51.6)	145 (52.3)	158 (50.5)
	≥25,000	360 (47.0)	435 (48.4)	132 (47.7)	155 (49.5)
Marital status					
	Married or living as married	483 (60.7)	546 (58.1)	209 (69.2)	224 (65.5)
	Not married or living as married	313 (39.3)	393 (41.9)	93 (30.8)	118 (34.5)
Cigarette smoking					
	Never smoked	651 (81.8)	– –	240 (79.5)	– –
	Current or former smoker	145 (18.2)	– –	62 (20.5)	– –
Years living in USA					
	Mean, years (interquartile range)	20 (14-29)	– –	20 (13-29)	– –
H2A visa					
	No	635 (93.1)	– –	246 (93.2)	– –
	Yes	47 (6.9)	– –	18 (6.8)	– –
Educational attainment					
	Less than high school	616 (77.3)	671 (71.3)	242 (80.4)	251 (73.6)
	High school diploma or higher	181 (22.7)	270 (28.7)	59 (19.6)	90 (26.4)
Child aged <5 years in household					
	No	513 (64.4)	616 (65.5)	178 (58.9)	205 (59.9)
	Yes	284 (35.6)	325 (34.5)	124 (41.1)	137 (40.1)
Household size					
	0-3 others	142 (17.8)	176 (18.7)	39 (12.9)	46 (13.5)
	4-5 others	340 (42.7)	408 (43.4)	130 (43.1)	149 (43.6)
	≥ 6 others	314 (39.5)	356 (37.9)	133 (44.0)	147 (43.0)
Persons per bedroom					
	0-2 persons	510 (64.1)	632 (67.2)	187 (62.3)	222 (65.3)
	>2-4 persons	269 (33.8)	289 (30.7)	93 (31.0)	98 (28.8)
	>4 persons	17 (2.1)	19 (2.0)	20 (6.7)	20 (5.9)
Washing machine in residence					
	No	283 (35.5)	– –	125 (41.4)	– –
	Yes	514 (64.5)	– –	177 (58.6)	– –
Field worker occupation					
	No	223 (28.3)	223 (28.1)	47 (15.7)	48 (15.8)
	Yes	566 (71.7)	570 (71.9	253 (84.3)	255 (84.2)
Commuted to work with non-household members (*N*=1,078)					
	No	515 (65.9)	– –	189 (63.6)	– –
	Yes	266 (34.1)	– –	108 (36.4)	– –
Commuted to work in employer provided bus (*N*=899)					
	No	593 (91.0)	– –	212 (85.8)	– –
	Yes	59 (9.0)	– –	35 (14.2)	– –
Number of people outside of household that you commute with (*N*=912)					
	0 others	444 (67.1)	– –	156 (62.4)	– –
	1-14 others	180 (27.2)	– –	70 (28)	– –
	≥15 others	38 (5.7)	– –	24 (9.6)	– –

Wearing a face covering when in proximity to others outside the home					– –
	Some of the time, rarely, or never	735 (92.2)	735 (92.2)	278 (92.1)	
	All or most of the time	62 (7.8)	62 (7.8)	24 (7.9)	– –
					– –
					– –

Table shows the prevalence of each of the exposures within the study populations stratified by carriage status. Dashes indicate variables not collected within the expanded study sample. Oral streptococcal carriage detection was defined as c_T values below 40 for *lytA* target.

Table S3: Detection of pneumococcal carriage among participants exposed to varying levels of household crowding, stratified according to the presence of a child aged <5 years in the household.

Characteristic		Any pneumococcal carriage				Higher-density pneumococcal carriage			
		Primary study population		*Expanded study population*		*Primary study population*		*Expanded study population*	
		No children aged <5 years in household	Child aged <5 years in household	No children aged <5 years in household	Child aged <5 years in household	No children aged <5 years in household	Child aged <5 years in household	No children aged <5 years in household	Child aged <5 years in household
Household size									
	0-3 others	8/161 (5.0)	1/20 (5.0)	10/194 (5.2)	2/28 (7.1)	2/161 (1.2)	1/20 (5.0)	3/194 (1.5)	2/28 (7.1)
	4-5 others	32/348 (9.2)	14/122 (11.5)	38/420 (9.0)	16/137 (11.7)	11/348 (3.2)	9/122 (7.4)	13/420 (3.1)	10/137 (7.3)
	≥6 others	18/181 (9.9)	30/266 (11.3)	20/206 (9.7)	31/297 (10.4)	8/181 (4.4)	16/266 (6.0)	9/206 (4.4)	16/297 (5.4)
Persons per bedroom									
	≤2 persons	32/497 (6.4)	21/200 (10.5)	41/617 (6.6)	24/237 (10.1)	7/497 (1.4)	13/200 (6.5)	10/617 (1.6)	14/237 (5.9)
	>2-4 persons	20/173 (11.6)	20/189 (10.6)	21/182 (11.5)	21/205 (10.2)	11/173 (6.4)	10/189 (5.3)	12/182 (6.6)	11/205 (5.4)
	≥4 persons	4/18 (22.2)	4/19 (21.1)	4/19 (21.1)	4/20 (20.0)	2/18 (11.1)	3/19 (15.8)	2/19 (10.5)	3/20 (15.0)

Table S4: Detection of pneumococcal carriage among participants exposed to varying levels of commuting environments, stratified according to the presence of household crowding.

Characteristic		Any pneumococcal carriage<br>*Primary study population*		Higher-density pneumococcal carriage<br>*Primary study population*	
		Uncrowded household (≤2 persons per bedroom)	Crowded household (>2 persons per bedroom)	Uncrowded household (≤2 persons per bedroom)	Crowded household (>2 persons per bedroom)
Commuted to work with non-household members (*N*=1,078)					
	No	34/473 (7.2)	25/231 (10.8)	15/473 (3.2)	12/231 (5.2)
	Yes	18/213 (8.5)	22/158 (13.9)	5/213 (2.3)	13/158 (8.2)
Commuted to work in employer provided bus (*N*=899)					
	No	38/537 (7.1)	30/267 (11.2)	18/537 (3.4)	14/267 (5.2)
	Yes	4/29 (13.8)	10/65 (15.4)	1/29 (3.4)	5/65 (7.7)
Number of people outside of household that you commute with (*N*=912)					
	0 others	26/398 (6.5)	22/202 (10.9)	14/398 (3.5)	10/202 (5.0)
	1-14 others	14/156 (9.0)	12/93 (12.9)	4/156 (2.6)	6/93 (6.5)
	≥15 others	2/18 (11.1)	8/44 (18.2)	1/18 (5.6)	5/44 (11.4)

Table S5: Associations of participant characteristics with detection of other *lytA*-positive oral Streptococcal carriage within the primary and expanded study populations.

Characteristics		Odds ratio (95% confidence interval)	
		Primary study population	*Expanded study population*
Age			
	18-29	ref.	ref.
	30-39	1.08 (0.74, 1.57)	1.09 (0.77, 1.55)
	40-49	1.24 (0.86, 1.80)	1.18 (0.84, 1.66)
	≥50	0.80 (0.53, 1.19)	0.84 (0.58, 1.21)
Sex			
	Female	ref.	ref.
	Male	0.9 (0.69, 1.17)	0.92 (0.71, 1.18)
Country of birth			
	Mexico or other	ref.	ref.
	United States	0.87 (0.58, 1.33)	0.97 (0.69, 1.36)
Language spoken at home			
	English	ref.	ref.
	Spanish	0.94 (0.50, 1.75)	0.89 (0.57, 1.38)
	Indigenous	2.35 (1.13, 4.86)	2.17 (1.21, 3.89)
Household income			
	< $25,000	ref.	ref.
	>= $25,000	1.04 (0.79, 1.38)	1.08 (0.83, 1.40)
Marital status			
	Not married or living as married	ref.	ref.
	Married or living as married	1.59 (1.19, 2.12)	1.45 (1.11, 1.88)
Cigarette smoking			
	Never smoked	ref.	– –
	Current or former smoker	1.17 (0.84, 1.64)	– –
Years living in USA			
	<5 years	ref.	– –
	5-18 years	0.59 (0.36, 0.95)	– –
	≥18 years	0.63 (0.41, 0.98)	– –
H2A visa			
	No	ref.	– –
	Yes	0.89 (0.50, 1.60)	– –
Educational attainment			
	Less than high school	ref.	ref.
	High school diploma or higher	0.88 (0.63, 1.23)	0.96 (0.72, 1.28)
Child aged <5 years in household			
	No	ref.	ref.
	Yes	1.31 (1.00, 1.73)	1.3 (1.01, 1.69)
Household size			
	0-3 others	ref.	ref.
	4-5 others	1.44 (0.95, 2.17)	1.43 (0.98, 2.09)
	≥ 6 others	1.58 (1.05, 2.40)	1.58 (1.07, 2.31)
Persons per bedroom			
	0-2 persons	ref.	ref.
	>2-4 persons	0.92 (0.69, 1.24)	0.93 (0.70, 1.23)
	>4 persons	2.95 (1.49, 5.83)	2.69 (1.39, 5.21)
Washing machine in residence			
	No	ref.	– –
	Yes	0.82 (0.62, 1.08)	– –
Field worker occupation			
	No	ref.	– –
	Yes	2.03 (1.43, 2.88)	– –
Commuted to work with non-household members (*N*=1,078)			
	No	ref.	– –
	Yes	1.05 (0.79, 1.39)	– –
Commuted to work in employer provided bus (*N*=899)			
	No	ref.	– –
	Yes	1.67 (1.06, 2.62)	– –
Number of people outside of household that you commute with (*N*=912)			
	0 others	ref.	– –
	1-14 others	0.99 (0.71, 1.40)	– –
	≥15 others	1.80 (1.03, 3.12)	– –
Wearing a face covering when in proximity to others outside the home			

	All or most of the time	ref.	– –
	Some of the time, rarely, or never	1.10 (0.67, 1.80)	– –

Odds ratios are computed via conditional logistic regression models stratified by recruitment venue and SARS-CoV-2 infection status.

Table S6: Association of participant characteristics with *lytA* and *piaB* c_T values among pneumococcal carriers.

		Target: *lytA*		Target: *piaB*	
**Characteristic**		*Mean difference (95% CI)*	*Adjusted mean Difference (95% CI)*	*Mean difference (95% CI)*	*Adjusted mean difference (95% CI)*
Sex					
	Female	ref.	ref.	ref.	ref.
	Male	-0.50 (-2.44, 1.42)	-0.10 (-2.08, 1.86)	-1.51 (-3.22, 0.19)	-1.46 (-3.12, 0.22)
Language spoken at home					
	English or Spanish	ref.	ref.	ref.	ref.
	Indigenous	0.06 (-2.08, 2.18)	0.00 (-2.31, 2.25)	-0.64 (-2.56, 1.25)	0.94 (-1.01, 2.85)
Child aged <5 years in household					
	No	ref.	ref.	ref.	ref.
	Yes	1.22 (-0.71, 3.13)	1.53 (-0.51, 3.56)	-2.04 (-3.73, -0.36)	-2.23 (-3.95, -0.49)
Persons per bedroom					
	0-2 people	ref.	ref.	ref.	ref.
	>2 people	-0.18 (-2.10, 1.73)	-0.57 (-2.64, 1.50)	-2.44 (-4.11, -0.80)	-2.19 (-3.94, -0.43)
Field worker occupation					
	No	ref.	ref.	ref.	ref.
	Yes	1.48 (0.96–2.30)	0.54 (-2.41, 3.49)	2.00 (1.06–3.79)	-0.62 (-3.09, 1.88)
SARS-CoV-2 infection					
	Negative	ref.	ref.	ref.	ref.
	Positive	0.52 (-1.67, 2.68)	0.35 (-1.91, 2.59)	-1.24 (-3.18, 0.69)	-1.45 (-3.35, 0.45)
Regular handwashing after touching objects outside the home					
	All or most of the time	ref.	ref.	ref.	ref.
	Some of the time, rarely, or never	3.25 (-0.81, 7.28)	4.41 (0.17, 8.62)	1.30 (-2.38, 4.95)	1.91 (-1.68, 5.49)
Wearing a face covering when in proximity to others outside the home					
	All or most of the time	ref.	ref.	ref.	ref.
	Some of the time, rarely, or never	-2.91 (-6.46, 0.62)	-4.10 (-7.87, -0.33)	-1.53 (-4.75, 1.65)	-1.80 (-5.00, 1.37)

SARS-CoV-2: Severe acute respiratory syndrome coronavirus 2, as detected by clinical transcription-mediated amplification testing from oropharyngeal specimens. In analyses including data from the expanded study population, there were no significant mean between-group differences in *lytA* c_T values. Significant mean between-group differences (MD) in *piaB* c_T values were apparent for crowded vs. uncrowded households (MD= -2.08 [-3.85, -0.33]; aMD -2.01 [-3.86, -0.19]) and households with or without children aged <5 years (MD = -2.17 [-3.95, -0.41]; aMD= -2.32 [-4.10, -0.53]).

Table S7. Associations of pneumococcal carriage with symptoms in last 2 weeks among study participants without SARS-CoV-2 infection.

Symptom			Association with pneumococcal carriage		Association with higher-density pneumococcal carriage	
		No pneumococcal carriage detected	*Pneumococcal carriage detected*	*OR (95% CI)*	*Pneumococcal carriage detected*	*OR (95% CI)*
		N=885	N=75		N=30	
Any symptom						
	Symptom absent	683 (77.2)	61 (81.3)	ref.	24 (80)	ref.
	Symptom present	202 (22.8)	14 (18.7)	0.78 (0.43,1.43)	6 (20)	0.84 (0.34,2.11)
Respiratory symptom						
	Symptom absent	733 (82.8)	65 (86.7)	ref.	26 (86.7)	ref.
	Symptom present	152 (17.2)	10 (13.3)	0.75 (0.37,1.49)	4 (13.3)	0.74 (0.25,2.17)
Dry cough						
	Symptom absent	853 (96.4)	71 (94.7)	ref.	28 (93.3)	ref.
	Symptom present	32 (3.6)	4 (5.3)	1.51 (0.52,4.39)	2 (6.7)	1.91 (0.44,8.35)
Productive cough						
	Symptom absent	846 (95.6)	72 (96)	ref.	28 (93.3)	ref.
	Symptom present	39 (4.4)	3 (4)	0.92 (0.28,3.05)	2 (6.7)	1.56 (0.36,6.82)
Pressure in ears						
	Symptom absent	874 (98.8)	72 (96)	ref.	29 (96.7)	ref.
	Symptom present	11 (1.2)	3 (4)	3.35 (0.91,12.27)	1 (3.3)	2.76 (0.34,22.18)
Blocked nose						
	Symptom absent	849 (95.9)	72 (96)	ref.	28 (93.3)	ref.
	Symptom present	36 (4.1)	3 (4)	1.00 (0.30,3.36)	2 (6.7)	1.71 (0.38,7.57)
Runny nose						
	Symptom absent	838 (94.7)	70 (93.3)	ref.	27 (90)	ref.
	Symptom present	47 (5.3)	5 (6.7)	1.30 (0.50,3.38)	3 (10)	2.01 (0.58,6.96)
Sneezing						
	Symptom absent	816 (92.2)	72 (96)	ref.	29 (96.7)	ref.
	Symptom present	69 (7.8)	3 (4)	0.50 (0.15,1.62)	1 (3.3)	0.41 (0.05,3.04)
Watery eyes						
	Symptom absent	844 (95.4)	74 (98.7)	ref.	30 (100)	– –
	Symptom present	41 (4.6)	1 (1.3)	0.28 (0.04,2.08)	0 (0)	– –
Hoarse voice						
	Symptom absent	857 (96.8)	73 (97.3)	ref.	29 (96.7)	ref.
	Symptom present	28 (3.2)	2 (2.7)	0.86 (0.20,3.71)	1 (3.3)	1.06 (0.14,8.19)
Self-reported fever						
	Symptom absent	865 (97.7)	72 (96)	ref.	27 (90)	ref.
	Symptom present	20 (2.3)	3 (4)	1.85 (0.53,6.43)	3 (10)	4.95 (1.36,18.02)
Sweating						
	Symptom absent	863 (97.5)	72 (96)	ref.	27 (90)	ref.
	Symptom present	22 (2.5)	3 (4)	1.67 (0.49,5.76)	3 (10)	4.47 (1.24,16.10)
Chills						
	Symptom absent	851 (96.2)	71 (94.7)	ref.	27 (90)	ref.
	Symptom present	34 (3.8)	4 (5.3)	1.45 (0.49,4.23)	3 (10)	2.84 (0.81,10.01)
Headache						
	Symptom absent	795 (89.8)	66 (88)	ref.	26 (86.7)	ref.
	Symptom present	90 (10.2)	9 (12)	1.22 (0.59,2.53)	4 (13.3)	1.36 (0.46,4.01)
Tickle in throat						
	Symptom absent	856 (96.7)	72 (96)	ref.	29 (96.7)	ref.
	Symptom present	29 (3.3)	3 (4)	1.25 (0.37,4.20)	1 (3.3)	1.02 (0.13,7.77)
Sore throat						
	Symptom absent	817 (92.3)	72 (96)	ref.	28 (93.3)	ref.
	Symptom present	68 (7.7)	3 (4)	0.50 (0.15,1.65)	2 (6.7)	0.86 (0.2,3.71)
Myalgia						
	Symptom absent	836 (94.5)	73 (97.3)	ref.	28 (93.3)	ref.

	Symptom present	49 (5.5)	2 (2.7)	0.47 (0.11,1.99)	2 (6.7)	1.23 (0.28,5.36)
Chest pain	Symptom absent	871 (98.4)	74 (98.7)	ref.	29 (96.7)	ref.
	Symptom present	14 (1.6)	1 (1.3)	0.85 (0.11,6.61)	1 (3.3)	2.16 (0.27,17.04)
Sinus pressure	Symptom absent	876 (99)	74 (98.7)	ref.	30 (100)	– –
	Symptom present	9 (1)	1 (1.3)	1.34 (0.17,10.68)	0 (0)	– –
Swollen glands	Symptom absent	873 (98.6)	74 (98.7)	ref.	30 (100)	– –
	Symptom present	12 (1.4)	1 (1.3)	0.98 (0.13,7.65)	0 (0)	– –
Loss of appetite	Symptom absent	870 (98.3)	73 (97.3)	ref.	28 (93.3)	ref.
	Symptom present	15 (1.7)	2 (2.7)	1.63 (0.36,7.34)	2 (6.7)	4.25 (0.91,19.84)
Difficulty breathing	Symptom absent	873 (98.6)	72 (96)	ref.	27 (90)	ref.
	Symptom present	12 (1.4)	3 (4)	3.07 (0.85,11.15)	3 (10)	8.12 (2.16,30.50)
Wheeze	Symptom absent	876 (99)	75 (100)	– –	30 (100)	– –
	Symptom present	9 (1)	0 (0)	– –	0 (0)	– –
Shortness of breath	Symptom absent	881 (99.5)	75 (100)	– –	30 (100)	– –
	Symptom present	4 (0.5)	0 (0)	– –	0 (0)	– –
Diarrhea	Symptom absent	860 (97.2)	75 (100)	– –	30 (100)	– –
	Symptom present	25 (2.8)	0 (0)	– –	0 (0)	– –
Nausea	Symptom absent	861 (97.3)	73 (97.3)	ref.	30 (100)	– –
	Symptom present	24 (2.7)	2 (2.7)	1.00 (0.23, 4.33)	0 (0)	– –
Stomach pain	Symptom absent	855 (96.6)	74 (98.7)	ref.	30 (100)	– –
	Symptom present	30 (3.4)	1 (1.3)	0.39 (0.05, 2.90)	0 (0)	– –
Difficulty thinking	Symptom absent	873 (98.6)	74 (98.7)	ref.	30 (100)	– –
	Symptom present	12 (1.4)	1 (1.3)	0.98 (0.13, 7.61)	0 (0)	– –
Fatigue	Symptom absent	830 (93.8)	71 (94.7)	ref.	29 (96.7)	ref.
	Symptom present	55 (6.2)	4 (5.3)	0.86 (0.30, 2.44)	1 (3.3)	0.52 (0.07,3.90)
Loss of taste	Symptom absent	874 (98.8)	75 (100)	– –	30 (100)	– –
	Symptom present	11 (1.2)	0 (0)	– –	0 (0)	– –
Loss of smell	Symptom absent	876 (99)	75 (100)	– –	30 (100)	– –
	Symptom present	9 (1)	0 (0)	– –	0 (0)	– –
Pain in eyes	Symptom absent	866 (97.9)	75 (100)	– –	30 (100)	– –
	Symptom present	19 (2.1)	0 (0)	– –	0 (0)	– –

Adjusted odds ratios are computed via conditional logistic regression models stratified by recruitment venue and adjusted for age and sex. Respiratory symptoms include dry cough, productive cough, blocked nose, runny nose, sneezing, hoarse voice, tickle in throat, sore throat, sinus pressure, difficulty breathing, wheeze, and shortness of breath. In analyses including the expanded study population, no symptoms were significantly associated with pneumococcal carriage; symptoms associated with higher-density pneumococcal carriage included self-reported fever (OR=4.25 [1.20-15.13]; aOR=4.11 [1.15-14.70]), sweats (OR=3.48 [1.00-12.09]; aOR=3.56 [1.01-12.50]), and difficulty breathing (OR=5.64 [1.58-20.11]; aOR=5.63 [1.57-20.21]).

Table S8. Associations of pneumococcal carriage with symptoms in last 2 weeks among all study participants.

Symptom			**Association with pneumococcal carriage**		**Association with higher-density pneumococcal carriage**	
		No pneumococcal carriage detected	*Pneumococcal carriage detected*	*OR (95% CI)*	*Higher-density carriage detected*	*Unadjusted OR (95% CI)*
		N=991	N=102		N=46	
Any symptom						
	Symptom absent	729 (73.6)	72 (70.6)	ref.	31 (67.4)	ref.
	Symptom present	262 (26.4)	30 (29.4)	1.08 (0.68, 1.70)	15 (32.6)	1.21 (0.64, 2.30)
Respiratory symptom						
	Symptom absent	786 (79.3)	80 (78.4)	ref.	37 (80.4)	ref.
	Symptom present	205 (20.7)	22 (21.6)	0.98 (0.59, 1.62)	9 (19.6)	0.83 (0.39, 1.75)
Dry cough						
	Symptom absent	936 (94.5)	92 (90.2)	ref.	41 (89.1)	ref.
	Symptom present	55 (5.5)	10 (9.8)	1.74 (0.86, 3.54)	5 (10.9)	1.88 (0.71, 4.97)
Productive cough						
	Symptom absent	933 (94.1)	94 (92.2)	ref.	42 (91.3)	ref.
	Symptom present	58 (5.9)	8 (7.8)	1.26 (0.58, 2.73)	4 (8.7)	1.35 (0.46, 3.93)
Pressure in ears						
	Symptom absent	974 (98.3)	96 (94.1)	ref.	43 (93.5)	ref.
	Symptom present	17 (1.7)	6 (5.9)	3.28 (1.26, 8.56)	3 (6.5)	3.37 (0.94, 12.07)
Blocked nose						
	Symptom absent	937 (94.6)	96 (94.1)	ref.	43 (93.5)	ref.
	Symptom present	54 (5.4)	6 (5.9)	0.97 (0.40, 2.34)	3 (6.5)	1.02 (0.3, 3.43)
Runny nose						
	Symptom absent	925 (93.3)	94 (92.2)	ref.	43 (93.5)	ref.
	Symptom present	66 (6.7)	8 (7.8)	1.11 (0.51, 2.39)	3 (6.5)	0.86 (0.26, 2.88)
Sneezing						
	Symptom absent	906 (91.4)	96 (94.1)	ref.	44 (95.7)	ref.
	Symptom present	85 (8.6)	6 (5.9)	0.63 (0.27, 1.49)	2 (4.3)	0.45 (0.11, 1.87)
Watery eyes						
	Symptom absent	938 (94.7)	99 (97.1)	ref.	45 (97.8)	ref.
	Symptom present	53 (5.3)	3 (2.9)	0.47 (0.14, 1.54)	1 (2.2)	0.32 (0.04, 2.38)
Hoarse voice						
	Symptom absent	950 (95.9)	97 (95.1)	ref.	43 (93.5)	ref.
	Symptom present	41 (4.1)	5 (4.9)	1.06 (0.4, 2.77)	3 (6.5)	1.35 (0.40, 4.59)
Self-reported fever						
	Symptom absent	950 (95.9)	91 (89.2)	ref.	39 (84.8)	ref.
	Symptom present	41 (4.1)	11 (10.8)	2.55 (1.25, 5.2)	7 (15.2)	3.61 (1.5, 8.71)
Sweating						
	Symptom absent	954 (96.3)	94 (92.2)	ref.	41 (89.1)	ref.
	Symptom present	37 (3.7)	8 (7.8)	1.98 (0.89, 4.43)	5 (10.9)	2.7 (1.00, 7.34)
Chills						
	Symptom absent	934 (94.2)	84 (82.4)	ref.	39 (84.8)	ref.
	Symptom present	57 (5.8)	12 (11.8)	1.98 (1.01, 3.88)	7 (15.2)	2.52 (1.06, 6.00)
Headache						
	Symptom absent	867 (87.5)	84 (82.4)	ref.	38 (82.6)	ref.
	Symptom present	124 (12.5)	18 (17.6)	1.41 (0.81, 2.44)	8 (17.4)	1.34 (0.61, 2.95)
Tickle in throat						
	Symptom absent	951 (96)	98 (96.1)	ref.	45 (97.8)	ref.
	Symptom present	40 (4)	4 (3.9)	0.9 (0.31, 2.58)	1 (2.2)	0.47 (0.06, 3.5)
Sore throat						
	Symptom absent	901 (90.9)	93 (91.2)	ref.	42 (91.3)	ref.
	Symptom present	90 (9.1)	9 (8.8)	0.88 (0.43, 1.82)	4 (8.7)	0.83 (0.29, 2.38)
Myalgia						
	Symptom absent	910 (91.8)	92 (90.2)	ref.	41 (89.1)	ref.

	Symptom present	81 (8.2)	10 (9.8)	1.09 (0.54, 2.21)	5 (10.9)	1.16 (0.44, 3.07)
Chest pain	Symptom absent	969 (97.8)	99 (97.1)	ref.	45 (97.8)	ref.
	Symptom present	22 (2.2)	3 (2.9)	1.20 (0.35, 4.12)	1 (2.2)	0.84 (0.11, 6.4)
Sinus pressure	Symptom absent	978 (98.7)	99 (97.1)	ref.	45 (97.8)	ref.
	Symptom present	13 (1.3)	3 (2.9)	2.06 (0.57, 7.38)	1 (2.2)	1.44 (0.18, 11.28)
Swollen glands	Symptom absent	976 (98.5)	100 (98)	ref.	46 (100)	– –
	Symptom present	15 (1.5)	2 (2)	1.26 (0.28, 5.6)	0 (0)	– –
Loss of appetite	Symptom absent	959 (96.8)	97 (95.1)	ref.	42 (91.3)	ref.
	Symptom present	32 (3.2)	5 (4.9)	1.38 (0.52, 3.65)	4 (8.7)	2.42 (0.81, 7.26)
Difficulty breathing	Symptom absent	966 (97.5)	94 (92.2)	ref.	41 (89.1)	ref.
	Symptom present	25 (2.5)	8 (7.8)	3.01 (1.31, 6.92)	5 (10.9)	4.14 (1.50, 11.47)
Wheeze	Symptom absent	979 (98.8)	100 (98)	ref.	46 (100)	– –
	Symptom present	12 (1.2)	2 (2)	1.48 (0.33, 6.74)	0 (0)	– –
Shortness of breath	Symptom absent	976 (98.5)	96 (94.1)	ref.	43 (93.5)	ref.
	Symptom present	15 (1.5)	6 (5.9)	3.66 (1.38, 9.72)	3 (6.5)	3.86 (1.06, 13.96)
Diarrhea	Symptom absent	954 (96.3)	99 (97.1)	ref.	44 (95.7)	ref.
	Symptom present	37 (3.7)	3 (2.9)	0.69 (0.21, 2.31)	2 (4.3)	0.98 (0.23, 4.25)
Nausea	Symptom absent	958 (96.7)	97 (95.1)	ref.	44 (95.7)	ref.
	Symptom present	33 (3.3)	5 (4.9)	1.34 (0.51, 3.55)	2 (4.3)	1.12 (0.26, 4.86)
Stomach pain	Symptom absent	948 (95.7)	99 (97.1)	ref.	45 (97.8)	ref.
	Symptom present	43 (4.3)	3 (2.9)	0.62 (0.19, 2.03)	1 (2.2)	0.43 (0.06, 3.2)
Difficulty thinking	Symptom absent	974 (98.3)	101 (99)	ref.	46 (100)	– –
	Symptom present	17 (1.7)	1 (1)	0.56 (0.07, 4.22)	0 (0)	– –
Fatigue	Symptom absent	914 (92.2)	90 (88.2)	ref.	40 (87)	ref.
	Symptom present	77 (7.8)	12 (11.8)	1.46 (0.76, 2.81)	6 (13)	1.57 (0.64, 3.85)
Loss of taste	Symptom absent	963 (97.2)	99 (97.1)	ref.	45 (97.8)	ref.
	Symptom present	28 (2.8)	3 (2.9)	0.95 (0.28, 3.18)	1 (2.2)	0.66 (0.09, 5)
Loss of smell	Symptom absent	965 (97.4)	99 (97.1)	ref.	45 (97.8)	ref.
	Symptom present	26 (2.6)	3 (2.9)	1.03 (0.30, 3.47)	1 (2.2)	0.72 (0.1, 5.46)
Pain in eyes	Symptom absent	969 (97.8)	99 (97.1)	ref.	45 (97.8)	ref.
	Symptom present	22 (2.2)	3 (2.9)	1.27 (0.37, 4.33)	1 (2.2)	1.55 (0.53, 4.54)

Adjusted odds ratios are computed via conditional logistic regression models stratified by recruitment venue and adjusted for age and sex. Respiratory symptoms include dry cough, productive cough, blocked nose, runny nose, sneezing, hoarse voice, tickle in throat, sore throat, sinus pressure, difficulty breathing, wheeze, and shortness of breath. In analyses including the expanded study population, symptoms significantly associated with pneumococcal carriage included painful ear preasure (OR=3.36 [1.38-8.16]; aOR=3.38 [1.38-8.24]), self-reported fever (OR=2.94 [1.55-5.57]; aOR 2.97 [1.56-5.65]), sweats (OR=2.1 [1.02-4.3]; aOR=2.06 [1-4.23]); chills (OR=1.85 [1-3.42]; aOR=1.85 [1-3.42]), swollen glands (OR=2.69 [0.98-7.37]; aOR=2.93 [1.06-8.09]), difficultly breathing (OR=3.08 [1.47-6.47]; aOR=3.06 [1.45-6.44]), and shortness of breath (OR=3.51 [1.44-8.57]; aOR=3.6 [1.47-8.85]). Symptoms associated with higher-density pneumococcal carriage included self-reported fever (OR=4.33 [1.96-9.54]; aOR=4.35 [1.96-9.67]), sweats (OR=2.81 [1.13-6.97]; aOR=2.86 [1.14-7.13]), chills (OR= 2.37 [1.06-5.28]; aOR=2.43 [1.08-5.44]), and difficulty breathing (OR=4.15 [1.64-10.48]; aOR=4.1 [1.61-10.41]).

Table S9: Association of symptoms with *lytA* and *piaB* c_T values, among SARS-CoV-2 negative participants.

Symptom	Mean c_T difference (95% CI)	
	lytA	*piaB*
Any symptom	-1.15 (-3.94, 1.65)	0.19 (-2.33, 2.70)
Respiratory symptoms	-1.01 (-4.12, 2.10)	0.73 (-2.06, 3.52)
Nonproductive cough	-1.96 (-7.24, 3.33)	0.25 (-4.50, 5.01)
Productive cough	1.08 (-5.00, 7.16)	-3.15 (-8.56, 2.26)
Pain or pressure in the ears	-2.69 (-8.74, 3.37)	-0.94 (-6.39, 4.51)
Blocked nose	-3.14 (-9.18, 2.91)	-2.57 (-7.99, 2.86)
Runny nose	-0.99 (-5.07, 3.08)	-0.21 (-3.87, 3.45)
Sneezing	-2.03 (-7.32, 3.25)	-2.10 (-6.83, 2.63)
Watery eyes	-3.77 (-14.15, 6.61)	3.93 (-5.36, 13.23)
Hoarseness	-2.90 (-10.28, 4.48)	0.36 (-6.28, 6.99)
Self-reported fever	-1.82 (-7.90, 4.25)	-2.08 (-7.52, 3.35)
Sweating	-1.82 (-7.90, 4.25)	-2.08 (-7.52, 3.35)
Chills	-3.29 (-8.55, 1.96)	-0.35 (-5.10, 4.40)
Headache	-2.33 (-5.95, 1.28)	0.21 (-3.06, 3.48)
Tickle in throat	-3.24 (-9.29, 2.80)	1.58 (-3.86, 7.03)
Sore throat	-2.68 (-7.95, 2.59)	-1.35 (-6.09, 3.39)
Myalgia	-1.34 (-8.75, 6.06)	-3.48 (-10.08, 3.11)
Chest pain	-5.04 (-15.40, 5.31)	-4.79 (-14.06, 4.49)
Sinus pain	-7.36 (-17.65, 2.93)	4.73 (-4.54, 14.01)
Swollen glands	-3.77 (-14.15, 6.61)	3.93 (-5.36, 13.23)
Loss of appetite	-0.15 (-7.56, 7.26)	-0.66 (-7.30, 5.97)
Difficulty breathing	-1.04 (-7.12, 5.05)	-4.63 (-9.99, 0.73)
Wheezing	– –	– –
Shortness of breath	– –	– –
Diarrhea	– –	– –
Nausea	-4.07 (-11.43, 3.28)	5.17 (-1.37, 11.72)
Stomach pain	-0.09 (-10.51, 10.32)	5.19 (-4.07, 14.46)
Trouble thinking	-0.09 (-10.51, 10.32)	5.19 (-4.07, 14.46)
Fatigue	-2.29 (-7.57, 2.99)	3.05 (-1.66, 7.76)
Loss of sense of taste	– –	– –
Loss of sense of smell	– –	– –
Pain or pressure in the eyes	– –	– –

Symptoms represent self-reported symptoms within the previous 2 weeks. Respiratory symptoms include dry cough, productive cough, blocked nose, runny nose, sneezing, hoarse voice, tickle in throat, sore throat, sinus pressure, difficulty breathing, wheeze, and shortness of breath.

Table S10: Association of symptoms with *lytA* and *piaB* c_T values, among all study participants.

Symptom	Mean c_T difference (95% CI)	
	lytA	*piaB*
Any symptom	-0.31 (-2.27, 1.64)	-0.72 (-2.65. 1.2)
Respiratory symptoms	-0.05 (-2.16, 2.05)	0.03 (-2.05, 2.11)
Nonproductive cough	-0.06 (-3.16, 3.05)	-0.42 (-3.49, 2.66)
Productive cough	2 (-1.23, 5.22)	-0.27 (-3.48, 2.94)
Pain or pressure in the ears	-0.56 (-4.39, 3.26)	-0.99 (-4.77, 2.79)
Blocked nose	-2 (-5.58, 1.58)	-2.13 (-5.66, 1.41)
Runny nose	1.21 (-1.77, 4.19)	1.57 (-1.37, 4.52)
Sneezing	-1.52 (-4.62, 1.57)	-2.17 (-5.22, 0.88)
Watery eyes	1.69 (-2.78, 6.17)	1.77 (-2.65, 6.19)
Hoarseness	-0.66 (-4.48, 3.17)	-1.06 (-4.83, 2.72)
Self-reported fever	0.21 (-2.59, 3)	-0.61 (-3.37, 2.15)
Sweating	1.26 (-1.98, 4.50)	0.51 (-2.70, 3.72)
Chills	-1.05 (-3.84, 1.74)	-0.72 (-3.48, 2.04)
Headache	-1.43 (-3.74, 0.87)	-0.87 (-3.16, 1.42)
Tickle in throat	-2.58 (-7.04, 1.88)	-0.49 (-4.93, 3.94)
Sore throat	0.15 (-2.74, 3.03)	-0.06 (-2.92, 2.79)
Myalgia	-0.22 (-3.11, 2.66)	-1.03 (-3.88, 1.82)
Chest pain	-1.1 (-5.58, 3.38)	-0.91 (-5.34, 3.52)
Sinus pain	2.23 (-2.74, 7.21)	4.16 (-0.71, 9.04)
Swollen glands	-0.37 (-4.85, 4.12)	-0.29 (-4.72, 4.15)
Loss of appetite	-2.96 (-7.42, 1.49)	-3.1 (-7.5, 1.29)
Difficulty breathing	-1.19 (-4.43, 2.05)	-2.2 (-5.39, 0.98)
Wheezing	1.23 (-4.5, 6.97)	1.79 (-3.87, 7.46)
Shortness of breath	1.45 (-2.36, 5.27)	0.19 (-3.59, 3.97)
Diarrhea	-5.23 (-10.89, 0.43)	-5.21 (-10.8, 0.38)
Nausea	-2.51 (-6.31, 1.29)	-0.15 (-3.93, 3.63)
Stomach pain	-1.84 (-6.82, 3.15)	-0.14 (-5.07, 4.8)
Trouble thinking	-1.74 (-7.47, 4)	-1.01 (-6.68, 4.66)
Fatigue	-1.06 (-3.85, 1.73)	-0.19 (-2.96, 2.57)
Loss of sense of taste	-4.14 (-9.08, 0.80)	-5.08 (-9.92, -0.23)
Loss of sense of smell	-4.14 (-9.08, 0.80)	-5.08 (-9.92, -0.23)
Pain or pressure in the eyes	-4.14 (-9.08, 0.80)	-5.08 (-9.92, -0.23)

Symptoms represent self-reported symptoms within the previous 2 weeks. Respiratory symptoms include dry cough, productive cough, blocked nose, runny nose, sneezing, hoarse voice, tickle in throat, sore throat, sinus pressure, difficulty breathing, wheeze, and shortness of breath.

Table S11: Distribution of risk factors among participants who reported speaking Indigenous languages at home and participants who did not report speaking Indigenous languages.

Characteristic		Participants, *n* (%)		
		Non-indigenous language	Indigenous language	p-value
		N=1,173	*N*=110	
Age range, years				<0.001
	18-29	300 (25.6)	44 (40)	
	30-39	274 (23.4)	32 (29.1)	
	40-49	303 (25.8)	24 (21.8)	
	≥50	296 (25.2)	10 (9.1)	
Country of birth				<0.001
	Mexico or other	949 (80.9)	108 (98.2)	
	United States	224 (19.1)	2 (1.8)	
Annual household income				<0.001
	<25,000	552 (49.2)	70 (76.9)	
	25,000-34,999	268 (23.9)	16 (17.6)	
	35,000-49,000	191 (17)	4 (4.4)	
	≥ 50,000	110 (9.8)	1 (1.1)	
Marital status				<0.001
	Married or living as married	682 (58.2)	88 (80.7)	
	Not married or living as married	490 (41.8)	21 (19.3)	
Cigarette smoking				0.051
	Never smoked	795 (80.4)	96 (88.1)	
	Current or former smoker	194 (19.6)	13 (11.9)	
Years living in United States				<0.001
	<5 years	74 (8.7)	31 (29.0)	
	5-18 years	218 (25.6)	47 (43.9)	
	≥18 years	559 (65.7)	29 (27.1)	
H-2A Visa				>0.9
	No	783 (93.1)	98 (93.3)	
	Yes	58 (6.9)	7 (6.7)	
Educational attainment				<0.001
	Never attended school	40 (3.4)	23 (21.1)	
	Some primary school	398 (33.9)	62 (56.9)	
	Primary school completed	235 (20)	16 (14.7)	
	Some high school	143 (12.2)	5 (4.6)	
	High school completed	357 (30.4)	3 (2.8)	
Child aged <5 years in household				<0.001
	No	773 (65.9)	48 (43.6)	
	Yes	400 (34.1)	62 (56.4)	
Household size				<0.001
	0-3 others	214 (18.3)	8 (7.3)	
	4-5 others	529 (45.1)	28 (25.5)	
	≥ 6 others	429 (36.6)	74 (67.3)	
Persons per bedroom				<0.001
	0-2 people	822 (70.1)	32 (29.9)	
	3-4 people	326 (27.8)	61 (57.0)	
	≥ 4 people	25 (2.1)	14 (13.1)	
Washing machine in residence				<0.001
	No	332 (33.5)	76 (69.7)	
	Yes	658 (66.5)	33 (30.3)	
Field worker occupation				<0.001
	No	268 (27.3)	2 (1.9)	
	Yes	715 (72.7)	104 (98.1)	
Workplace setting (*N*=898)				
	Indoors and outdoor	217(27.1)	2 (2.1)	<0.001
	Outdoors only	585 (72.9)	94 (97.9)	
Commuted to work with non-household members (*N*=1,078)				0.14
	No	641 (66.0)	63 (58.9)	
	Yes	330 (34.0)	44 (41.1)	
Commuted to work in employer provided bus (*N*=899)				
	No	718 (89.5)	87 (89.7)	>0.9
	Yes	84 (10.5)	10 (10.3)	
Number of people outside of household that you commute with (*N*=912)				0.8
	0 others	540 (66.2)	60 (62.5)	
	1-14 others	221 (27.1)	29 (30.2)	
	≥15 others	55 (6.7)	7(7.3)	
Wearing a face covering when in proximity to others outside the home				0.6
	Some of the time, rarely, or never	79 (8)	7 (6.4)	
	All or most of the time	911 (92)	102 (93.6)	

P-values are computed via Pearson's chi-squared test and Fisher's exact test.
